# Therapeutic Nanocarriers Inhibit Chemotherapy‐Induced Breast Cancer Metastasis

**DOI:** 10.1002/advs.202203949

**Published:** 2022-10-11

**Authors:** Tianyu Li, Tolulope Akinade, Jie Zhou, Hongxia Wang, Qisong Tong, Siyu He, Emily Rinebold, Luis E. Valencia Salazar, Divya Bhansali, Yiling Zhong, Jing Ruan, Jinzhi Du, Piero Dalerba, Kam W. Leong

**Affiliations:** ^1^ Department of Biomedical Engineering Columbia University New York NY 10027 USA; ^2^ Graduate Program in Cellular, Molecular and Biomedical Studies Vagelos College of Physicians and Surgeons Columbia University New York NY 10027 USA; ^3^ Department of Breast Oncology Affiliated Cancer Hospital and Institute of Guangzhou Medical University Guangzhou 510095 P. R. China; ^4^ School of Biomedical Sciences and Engineering Guangzhou International Campus South China University of Technology Guangzhou 511442 P. R. China; ^5^ Department of Pathology & Cell Biology Department of Medicine (Division of Digestive and Liver Diseases) Herbert Irving Comprehensive Cancer Center (HICCC) and Columbia Stem Cell Initiative (CSCI) Columbia University New York NY 10032 USA; ^6^ Department of Surgery (Division of Colorectal Surgery) Columbia University Medical Center New York NY 10032 USA; ^7^ Department of Systems Biology Columbia University Medical Center New York NY 10032 USA

**Keywords:** breast cancer, cell‐free nucleic acid, chemotherapy, metastasis, nanocarrier, scavenger

## Abstract

Chemotherapy, although effective against primary tumors, may promote metastasis by causing the release of proinflammatory factors from damaged cells. Here, polymeric nanoparticles that deliver chemotherapeutics and scavenge proinflammatory factors simultaneously to inhibit chemotherapy‐induced breast cancer metastasis are developed. The cationic nanoparticles can adsorb cell‐free nucleic acids (cfNAs) based on charge–charge interaction, which downregulates the expression of Toll‐like receptors and then reduces the secretion of inflammatory cytokines. Through in vitro structural optimization, cationic polyamidoamine (PAMAM) dendrimers modified with drug‐binding dodecyl groups and diethylethanolamine surface groups (PAMAM‐G3‐C12_5_‐DEEA_20_) exhibit the most desirable combination of nanoparticle size (≈140 nm), drug loading, cytotoxicity, cfNA binding, and anti‐inflammatory activity. In the mouse models of breast cancer metastasis, paclitaxel‐loaded nanoparticles reduce serum levels of cfNAs and inflammatory cytokines compared with paclitaxel treatment alone and inhibit both primary tumor growth and tumor metastasis. Additionally, no significant side effects are detected in the serum or major organs. These results provide a strategy to deliver chemotherapeutics to primary tumors while reducing the prometastatic effects of chemotherapy.

## Graphical abstract

Polymeric nanoparticles are developed, that deliver chemotherapeutics and scavenge damage‐associated molecular patterns simultaneously to inhibit chemotherapy‐induced breast cancer metastasis. The cationic nanoparticles can adsorb cell‐free nucleic acids based on charge–charge interaction, which downregulates the expression of Toll‐like receptors and then reduces the secretion of inflammatory cytokines.

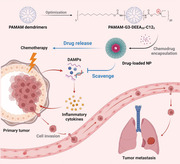

## 1 Introduction

Cancer is the leading cause of death in most countries,^[^ ^1^ ^]^ and among females, breast cancer is the most commonly diagnosed and has the second highest death rate.^[^ ^2^ ^]^ Many patients with early‐stage nonmetastatic breast cancer can be cured with a combination of surgery, chemotherapy, hormone therapy, and radiotherapy, but ≈50% of patients develop distant organ metastases, which are typically incurable.^[^ ^3^ ^]^ Chemotherapeutics such as paclitaxel (PTX) and doxorubicin are used in combination with surgery to treat primary breast cancer. Although these drugs inhibit primary tumor growth, growing evidence suggests that they may promote metastasis by causing elevated levels of proinflammatory cell‐free nucleic acids (cfNAs), which are released by damaged cells into the tumor microenvironment.^[^ ^4^ ^]^ Thus, new studies have started focusing on attenuating the prometastatic effects of chemotherapy.^[^ ^5^ ^]^

Elevated levels of cfNAs—single‐ and double‐stranded DNA and RNA—surrounding tumors and in circulation following chemotherapy have been associated with metastasis.^[^ ^6^ ^]^ cfNAs are damage‐associated molecular patterns (DAMPs) that induce chronic inflammation by activating Toll‐like receptors (TLRs);^[^ ^7^ ^]^ for example, dsRNA activates TLR3, ssRNA activates TLR8, and ssDNA activates TLR9. Activated TLRs upregulate MYD88 and NF‐*κ*B to induce the secretion of inflammatory cytokines,^[^ ^8^ ^]^ and TLR‐induced inflammation appears to promote tumor progression and metastasis.^[^ ^9^ ^]^ cfNAs are found at higher concentrations in the serum of metastatic breast cancer patients than in healthy individuals^[^ ^10^ ^]^ and are being explored as biomarkers for cancer diagnosis and prognosis;^[^ ^11^ ^]^ however, they are seldom recognized as therapeutic targets for preventing metastasis.

Our previous studies showed that cationic polyamidoamine (PAMAM) dendrimers can scavenge nucleic acids^[^ ^12^ ^]^ via electrostatic interactions. When used to treat inflammatory and autoimmune diseases, PAMAM scavenges cfNAs, inhibiting cfNA‐induced TLR activation and TLR‐induced inflammation.^[^ ^13^ ^]^ In pancreatic cancer and breast cancer models, PAMAM dendrimers inhibited metastasis by scavenging cfNAs.^[^ ^14^ ^]^ However, PAMAM has not been used to prevent chemotherapy‐induced metastasis, and its side effects limit its applications.

Encapsulation of chemotherapeutics by polymeric nanocarriers has been widely used strategy to enhance drug delivery,^[^ ^15^ ^]^ yielding greater drug stability and better tumor targeting.^[^ ^16^ ^]^ Here, we created a PAMAM‐based nanoparticle (NP) and optimized its structure to reduce PAMAM cytotoxicity while also achieving high drug loading and cfNA binding, allowing the delivery of a chemotherapeutic drug and inhibiting the prometastatic inflammation caused by chemotherapy. We synthesized and compared a large set of PAMAM derivatives with different numbers of dodecyl (C12) groups (to create amphiphilicity for NP formation and drug loading) and different numbers of dimethyl‐, diethyl‐, or dibutylethanolamine (DMEA, DEEA, and DBEA) surface groups (to reduce the positive surface charge density) and identified that PAMAM‐G3‐C12_5_‐DEEA_20_ provided the best combination of NP size (≈140 nm), drug loading, cytotoxicity, cfNA binding, and anti‐inflammatory activity in vitro. In the mouse models of metastatic breast cancer, these cationic NPs encapsulated PTX and delivered it to breast tumor tissues to inhibit primary tumor growth while also scavenging cfNAs and inhibiting PTX‐induced inflammation and metastasis.

## 2 Results

### 2.1 Optimizing Polyamidoamine Dendrimer Derivatives for Low Cytotoxicity and High Cell‐Free Nucleic Acid Binding

**Figure 1:**
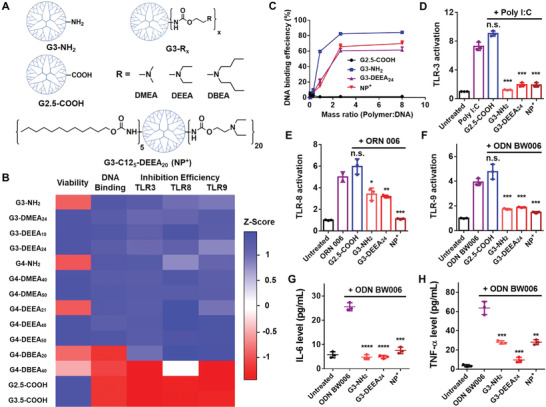
Structures and characterization of the PAMAM dendrimer derivatives. A) Top: Chemical structures of the PAMAM derivatives tested initially. Bottom: Structure of G3‐C12_5_‐DEEA_20_, which was selected after additional optimization for the preparation of the nanoparticles. “NP^+^” is used as an abbreviation for these cationic nanoparticles. B) Heatmap of the Z scores for the different attributes of the PAMAM derivatives. Higher Z scores (blue) indicate better cell viability, DNA binding, or TLR3/8/9 inhibition. C) DNA‐binding efficiency of different PAMAM derivatives and G3‐C12_5_‐DEEA_20_ nanoparticles at different polymer:DNA mass ratios. D–F) TLR3/8/9 activation assays showing inhibition of nucleic acid agonist‐induced TLR activation by the nanomaterials (polymers or nanoparticles). The DNA agonist used to activate HEK‐Blue hTLR cells is listed at the top of each panel. The mass ratio of nanomaterials to agonist was 2:1. G,H) IL‐6 and TNF‐*α* levels released by RAW 264.7 cells treated with the agonist ODN BW006 with or without the addition of nanomaterials. Comparisons were made with the agonist only group. *p < 0.05, **p < 0.01, ***p < 0.001, ****p < 0.0001; Student's *t*‐test. Data represent the mean ± SD.

We initially synthesized a set of 14 PAMAM dendrimer derivatives (listed in Table S1, Supporting Information; shown schematically in **Figure** **1**A) using the synthetic route shown in Figure S1A, Supporting Information. The chemical structures were characterized with ^1^H NMR, ^13^C NMR, ^1^H‐^1^H COSY, ^1^H‐^13^C HSQC, and ^1^H‐^13^C HMBC spectroscopy (Figure S1B–F, Supporting Information). To reduce the toxicity of PAMAM, which is due to its high positive surface charge density, the PAMAM amino groups were modified with *N*,*N*‐dialkylethanolamines (dimethyl‐, diethyl‐, and dibutylethanolamine—DMEA, DEEA, and DBEA, respectively) by esterification to shield some of the positive charges while leaving some amino groups unshielded to allow cfNA binding. Longer alkyl chains on these surface groups provide greater shielding but reduce cfNA binding. A series of PAMAM derivatives were synthesized based on the PAMAM generation 3 and 4 (G3 and G4) cores.

The PAMAM derivatives were characterized based on their cytotoxicity (IC_50_ value), DNA binding (EC_50_ value using salmon sperm DNA), and TLR inhibition (Table S1, Supporting Information). Inhibition of TLR activation was assessed with HEK‐Blue hTLR3/8/9 cells. TLR3, TLR8, and TLR9 were chosen for evaluation because they are activated by different types of nucleic acids: TLR3 by dsRNA, TLR8 by ssRNA, and TLR9 by ssDNA (Figure S1G, Supporting Information). PAMAM dendrimers with unmodified ‐NH_2_ groups are cationic and exhibit strong cfNA scavenging abilities but high toxicity (Figure 1B); PAMAM dendrimers modified with —COOH groups are anionic and exhibit low toxicity but weak cfNA scavenging properties. Modification of PAMAM with DBEA yielded strong cfNA scavenging but high toxicity. G4‐DEEA_40_ and G3‐DEEA_24_ exhibited the best combination of low cytotoxicity, strong cfNA scavenging, and TLR3/8/9 activity inhibition and were selected for further development.

### 2.2 Nanoparticles That Encapsulate Paclitaxel and Attenuate Cell‐Free Nucleic Acid‐Induced Toll‐Like Receptor Activation

G4‐DEEA_40_ and G3‐DEEA_24_ were further modified with different numbers of dodecyl (C12) groups to create amphiphilic polymers capable of forming NPs that encapsulate chemotherapeutics. These NPs exhibited similar zeta potentials of +55 to +65 mV (Table S2, Supporting Information). According to the TLR inhibition results, higher dodecyl group conjugation led to lower TLR inhibition efficiency (Figure S1H, Supporting Information). The G3‐C12_5_‐DEEA_20_ NP was chosen for further study because it exhibited high TLR inhibition, and its smaller size relative to the other NPs (142 nm vs 164–194 nm) (Table S2, Supporting Information) would be more favorable for tumor permeation and cellular uptake. The G3‐C12_5_‐DEEA_20_ NP showed a DNA binding capacity similar to that of hydrophilic dendrimeric polymers and inhibited nucleic acid agonist‐induced activation of TLRs 3, 8, and 9 (Figure 1C–F). Treatment of RAW 264.7 murine macrophages with CpG oligodeoxynucleotides (ODNs) activated TLR9 expression and induced the secretion of TNF‐*α* and IL‐6 (Figure 1G,H); however, addition of the NPs reduced the expression of these inflammatory cytokines.

**Figure 2:**
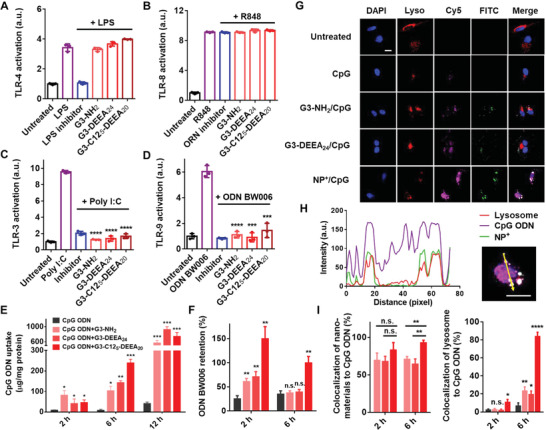
TLR inhibition and intracellular trafficking of nanoparticles. A,B) TLR4/8 activation assays with non‐NA agonists. G3‐C12_5_‐DEEA_20_ nanoparticles (NP^+^) did not inhibit non‐NA‐induced TLR activation. The mass ratio of the nanomaterial (polymers or nanoparticles) to agonist was 10:1. (C, D) TLR3/9 activation assays in which the nanomaterials were added 2 h before agonist treatment and were aspirated immediately before agonist addition. Comparisons were made with the agonist only group. E,F) Cellular uptake and retention of Cy5‐CpG ODNs with or without nanomaterial treatment. G) Fluorescence images showing MDA‐MB‐231 cellular uptake of CpG ODNs and nanomaterials at 6 h. Cell nuclei were stained with DAPI, lysosomes with LysoTracker Red, CpG ODNs with Cy5, and nanomaterials with FITC. Scale bar, 20 µm. H) Colocalization profiles of CpG ODN, NPs^+^, and lysosomes along the direction of the arrow in the image on the right. Scale bar, 20 µm. I) Quantification of colocalization percentages using Mander's overlap coefficient (MOC). Comparisons were made with the CpG ODN only group at the same time point unless indicated otherwise. *p < 0.05, **p < 0.01, ***p < 0.001, ****p < 0.0001; Student's *t*‐test. Data represent the mean ± SD.

We next tested whether the G3‐C12_5_‐DEEA_20_ NPs inhibited TLR activation specifically via cfNA binding. The NPs did not inhibit lipopolysaccharide (LPS)‐induced TLR4 activation in HEK‐Blue hTLR4 cells even at the high nanomaterial:LPS ratio of 10:1 (**Figure** **2**A). The NPs also did not inhibit TLR8 activation induced by the imidazoquinoline R848 in HEK‐Blue hTLR8 cells (Figure 2B). Moreover, the NPs showed no inhibitory effect on TLR activation after treatment with some common non‐nucleic acid TLR agonists, which is in contrast to their inhibition of nucleic acid agonist‐induced TLR activation.

We then studied whether the G3‐C12_5_‐DEEA_20_ NPs inhibited TLR activation by scavenging nucleic acids extracellularly or intracellularly. DNA binding experiments clearly indicated that the NPs could bind nucleic acids extracellularly, but we could not obtain direct evidence that they could also do so intracellularly. The NPs were first added to CpG ODN‐treated HEK‐Blue hTLR9 cells 2 h before, 2 h after, or at the same time as the agonist. The NPs inhibited TLR9 activation regardless of when they were added (Figure S2A, Supporting Information). The NPs were then added to HEK‐Blue hTLR3 and hTLR9 cells 2 h prior to the addition of the agonist and were removed by aspiration immediately before agonist addition. This treatment also resulted in the inhibition of TLR3 and TLR9 activation (Figure 2C,D), indicating that the NPs can act both extracellularly and intracellularly to inhibit TLR activation.

Next, CpG ODNs were labeled with Cy5 to monitor the cellular uptake and retention of cfNAs in the presence of the G3‐C12_5_‐DEEA_20_ NPs. The amount of CpG ODNs inside MDA‐MB‐231 human breast cancer cells was quantified based on Cy5 fluorescence (Figure 2E,F). Without NP treatment, there was little cellular uptake of CpG ODNs, but when the NPs were added, the cellular uptake of CpG ODNs increased. After a 12 h incubation, the media was replaced with fresh media to measure the cell retention of CpG ODNs. CpG ODN retention in NP‐treated cells was significantly higher than in the cells in the other treatment groups. The confocal laser scanning microscopy (CLSM) images directly revealed the cellular uptake of CpG ODN and NPs, which colocalized with lysosomes (Figure 2G–I, Figure S2B,C, Supporting Information). The cellular uptake of CpG ODN was the greatest upon treatment with G3‐C12_5_‐DEEA_20_ NPs. These results suggested that the NPs bound the CpG ODNs, trapping this cell‐free DNA (cfDNA) inside the cells.

### 2.3 G3‐C12_5_‐DEEA_20_ Nanoparticles Attenuate Paclitaxel/Damage‐Associated Molecular Pattern‐Induced Toll‐Like Receptor Activation by Scavenging Cell‐Free Nucleic Acids In Vitro

PTX is commonly used to treat breast cancer but may promote tumor metastasis by inducing the production of DAMPs.^[^ ^4a,b^ ^]^ We produced media containing PTX‐induced DAMPs from breast cancer cells by treating MDA‐MB‐231 cells with PTX for 6 h, replacing the media with PTX‐free Dulbecco's modified Eagle's medium (DMEM), culturing the cells for another 72 h, and collecting the resulting media. We refer to this media as PTX DAMP media. As a control, media was collected from cells treated with an equal volume of dimethyl sulfoxide (DMSO). The concentration of PTX used in the 6 h treatment step was optimized by incubating the cells with different amounts of Oregon Green‐labeled PTX. The amount of PTX remaining in the PTX DAMP media was determined based on fluorescence intensity measurements (Figure S3A–C, Supporting Information). PTX was not detected in the media when the cells were treated with ≤300 nM PTX. When MDA‐MB‐231 cells were cultured with the PTX DAMP media, no cytotoxicity was detected, also indicating a low level of PTX (Figure S3D, Supporting Information). The PTX DAMP media possessed higher levels of cfDNA and cfRNA than the control media and showed stronger activation of TLRs 3, 8, and 9, indicating the presence of proinflammatory DAMPs (Figure S3E–I, Supporting Information). These results indicated that PTX treatment elevated cfNA levels and increased TLR expression.

**Figure 3:**
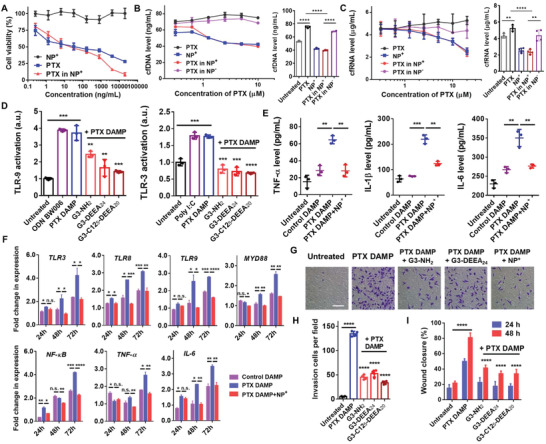
Nanoparticle‐mediated inhibition of paclitaxel/DAMP‐induced cancer cell migration and invasion. A) Viability of MDA‐MB‐231 cells treated with PTX only, G3‐C12_5_‐DEEA_20_ NPs (NP^+^), or PTX‐loaded NPs^+^ for 72 h. Concentrations indicate the amount of PTX in the PTX only or PTX‐NP^+^ samples. B,C) Cell‐free DNA (left) and cell‐free RNA (right) levels in the supernatants of MDA‐MB‐231 cells treated with PTX only, NPs^+^, PTX‐loaded NPs^+^, or PTX‐loaded NPs^−^ (anionic COOH‐modified NPs) for 72 h. D) TLR3/9 activation assays showed that the cationic NPs inhibited PTX DAMP‐induced TLR activation. PTX DAMPs—damage‐associated molecular patterns caused by paclitaxel treatment—were obtained by collecting the medium of MDA‐MB‐231 cells treated with PTX followed by PTX removal and incubation for 72 h. Comparisons were made with the PTX DAMP only group. E) TNF‐*α*, IL‐1*β*, and IL‐6 levels in THP‐1 cells treated with DAMPs and NPs^+^. F) Fold changes in the expression of genes in TLR‐related pathways. RNA was extracted from THP‐1 cells treated with DAMPs and NPs^+^ for 24, 48, or 72 h. Expression level ratios were normalized to those of the untreated groups. G) Transwell experiment showing invading MDA‐MB‐231 cells. Scale bar, 100 µm. H) Quantification of cell invasion. Cell numbers in each 20× field were counted. Comparisons were made with the PTX DAMP only group. I) Wound healing experiment showing migrating MDA‐MB‐231 cells and quantitation of the wound areas. Comparisons were made with the PTX DAMP only group. *p < 0.05, **p < 0.01, ***p < 0.001, ****p < 0.0001; Student's *t*‐test. Data represent the mean ± SD.

The encapsulation of PTX by the G3‐C12_5_‐DEEA_20_ NPs resulted in an increase in NP size from 141.8 ± 3.3 nm to 182.5 ± 0.9 nm but there was no significant change in zeta potential (58.2 ± 4.8 mV vs 58.6 ± 4.0 mV). Drug loading at a PTX:NP mass ratio of 30% yielded a loading efficiency of 96%. The release of PTX from the NPs was pH‐responsive (Figure S3J, Supporting Information). At pH 5.5, which mimics the acidic environment of endosome and lysosome, over 80% of PTX was released in 24 h. The toxicity of PTX‐loaded NPs to cancer cells was slightly higher than that of the free drug, likely because the NPs promoted cellular uptake of the drug (**Figure** **3**A). The NPs alone showed little cytotoxicity. Treatment with PTX alone resulted in increased cfDNA and cfRNA levels in the media (Figure 3B,C); treatment with NPs alone or PTX‐loaded NPs reduced the cfNA levels in the media. Anionic NPs synthesized by modifying PAMAM‐G2.5 with dodecylamine were used as a control. In the figures, the cationic G3‐C12_5_‐DEEA_20_ NPs are indicated as NP^+^ and the anionic NPs are denoted as NP^−^. These anionic NPs (145 nm diameter) were similar in size to the cationic NPs (142 nm) but had a zeta potential of −25 mV, which resulted in a low ability to scavenge cfNAs and weak inhibition of TLR activation (Figure S3K–Q, Supporting Information). Media from the cells treated with PTX‐loaded anionic NPs exhibited higher cfNA levels than media of cells treated with PTX‐loaded cationic NPs. These results indicated that the cationic NPs could scavenge cfNAs after loading PTX and that a positive surface charge is necessary for cfNA binding. The cationic NPs inhibited PTX DAMP‐induced TLR3 and TLR9 activation in HEK‐Blue hTLR cells (Figure 3D), confirming the cfNA scavenging ability of the cationic NPs.

### 2.4 G3‐C12_5_‐DEEA_20_ Nanoparticles Inhibit Toll‐Like Receptor‐Mediated Breast Cancer Cell Invasion and Migration In Vitro

The expression levels of the cytokines TNF‐*α*, IL‐1*β*, and IL‐6 were measured to assess the effects of PTX‐mediated DAMP‐induced inflammation (Figure 3E). THP‐1 human monocytes treated with PTX DAMP media showed significantly greater release of these cytokines than that observed from the untreated or control DAMP media treatment groups. The cytokine levels decreased after cotreatment with the G3‐C12_5_‐DEEA_20_ NPs. Further, the expression of TLR pathway genes was analyzed by qPCR (Figure 3F). Treatment with PTX DAMP media for 48 or 72 h resulted in greater expression of TLR3, TLR8, and TLR9 in THP‐1 cells and increased MYD88 expression. MYD88 activates NF‐*κ*B, upregulating the expression of TNF‐*α* and IL‐6. The expression of these genes was reduced by NP treatment, which indicated that the NPs inhibit the activation of these TLR pathways.

The effect of the NPs on the metastatic potential of cancer cells was assessed by investigating their effect on cancer cell invasion and migration. Cell invasion was evaluated with transwell experiments in Matrigel‐coated chambers (Figure 3G,H). Significantly more invading cells were observed in the PTX DAMP media treatment group than in the untreated group, and cotreatment with cationic NPs dramatically reduced the number of invading cells. Cancer cell migration was evaluated with a scratch wound healing assay. PTX DAMP media induced 80% wound closure after 48 h, whereas only 20% wound closure was observed with untreated MDA‐MB‐231 cells (Figure 3I, Figure S3R, Supporting Information). Adding cationic NPs to PTX DAMP media reduced wound closure from 80% to 30–40%. Together, these results indicated that PTX DAMP media promoted cancer cell invasion and migration and that the cationic NPs mitigated these effects.

### 2.5 Paclitaxel‐Loaded G3‐C12_5_‐DEEA_20_ Nanoparticles Inhibit Primary Tumor Progression In Vivo

**Figure 4:**
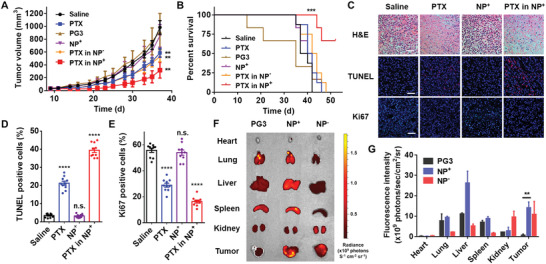
Effect of PTX‐loaded NPs on primary tumor progression and NP biodistribution in vivo. A,B) Primary tumor growth and Kaplan–Meier survival curves of 4T1 breast cancer‐bearing BALB/c mice treated with different therapeutic agents. Polymers (15 mg kg^−1^) and PTX (4.5 mg kg^−1^) were administered via i.p. injection three times a week. Mice were sacrificed after the tumor volumes exceeded 1000 mm^3^. C) Primary tumor sections stained with H&E, TUNEL, and Ki‐67. Scale bars, 50 µm. D,E) Quantification of TUNEL and Ki‐67^+^ cells in the primary tumor sections. F) Ex vivo imaging showing the biodistribution of different NPs 24 h after i.p. injection. Materials were labeled with Cy5. G) Quantification of the fluorescence intensity in different organs. Comparisons were made with the saline only group. *p < 0.05, **p < 0.01, ***p < 0.001, ****p < 0.0001; Student's *t*‐test. Data represent the mean ± SEM.

A murine breast cancer metastasis model was prepared to evaluate the antitumor efficacy of PTX‐loaded G3‐C12_5_‐DEEA_20_ NPs (Figure S4A, Supporting Information).^[^ ^17^ ^]^ Murine mammary carcinoma (4T1) cells were transfected with a plasmid encoding firefly luciferase Luc2P and injected subcutaneously into the mammary glands of female BALB/c mice. Treatments, including saline only, PTX, PAMAM‐G3, NP^+^, PTX‐loaded NP^+^, and PTX‐loaded NP^−^, were administered via i.p. injection. As above, NP^+^ indicates cationic G3‐C12_5_‐DEEA_20_ NPs, and NP^−^ indicates anionic NPs. Unencapsulated PTX inhibited tumor growth, but PTX‐loaded NPs^+^ inhibited tumor growth more efficiently. The tumor inhibition rate was 48.5% in the PTX group and 73.2% in the PTX‐loaded NP^+^ group (**Figure** **4**A, Figure S4B–D, Supporting Information). The improved antitumor efficiency was likely due to the NP‐mediated targeting and retention within tumor tissues. The results also showed that cationic NPs alone (PAMAM‐G3 and NPs^+^) could not inhibit primary tumor growth. Mice were sacrificed once the primary tumor volume exceeded 1000 mm^3^. The mice treated with PTX‐loaded NPs^+^ showed a significantly improved survival rate compared with untreated mice (Figure 4B).

Tumor tissues were collected after the mice were sacrificed. Formalin‐fixed paraffin‐embedded slides were stained with hematoxylin and eosin (H&E), TUNEL, and Ki‐67 to detect cell proliferation and apoptosis in the primary tumors (Figure 4C). PTX and PTX‐loaded NPs^+^ induced primary tumor cell apoptosis and necrosis, whereas NPs^+^ alone did not show clear antiproliferative activity. Higher percentages of TUNEL‐positive cells were observed in the PTX and PTX‐loaded NP^+^ groups, indicating the induction of apoptosis in primary tumor tissues by PTX and even more so by PTX‐loaded NPs^+^ (Figure 4D). Ki‐67 staining revealed the proliferation of tumor cells.^[^ ^18^ ^]^ Fewer Ki‐67^+^ cells were detected in the PTX and PTX‐loaded NP^+^ groups, indicating their efficacy against primary tumors (Figure 4E). The biodistribution of PAMAM‐G3, NPs^+^, and NPs^−^ in vivo was further studied with Cy5‐labeled nanomaterials. 24 h after i.p. injection, more NPs had accumulated in the tumor tissues than the hydrophilic PAMAM‐G3 polymer, indicating improved tumor targeting by the G3‐C12_5_‐DEEA_20_ NPs (Figure 4F,G).

### 2.6 Paclitaxel‐Loaded G3‐C12_5_‐DEEA_20_ Nanoparticles Inhibit Tumor Metastasis In Vivo

**Figure 5:**
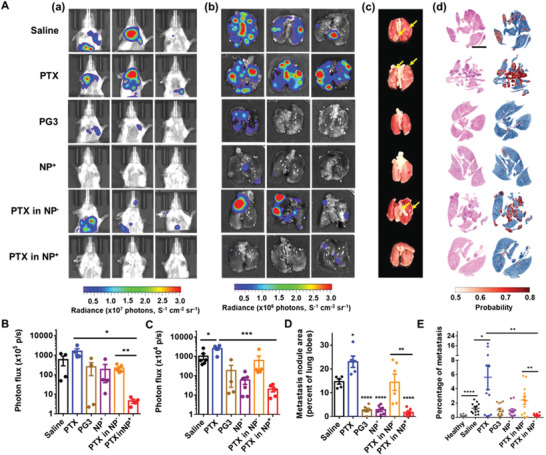
Effect of PTX‐loaded NPs on tumor metastasis. A) Images depicting tumor metastases in mice. a) Images of mice during week 6. Primary tumors were covered with black paper to shield their bright signals to better detect metastases. b) Ex vivo images of lungs collected immediately after the mice were sacrificed showing tumor metastasis signals. c) Images of lung lobes. Arrows indicate metastases. d) H&E‐stained lung sections and probability maps generated by a deep learning method that identifies metastatic tissue. The probability heatmap indicates the probability that each cell is metastatic. Scale bar, 2 mm. B) Total photon flux from in vivo images. C) Total photon flux from ex vivo images. D) Area analysis of the metastatic nodules on lung lobes. Comparisons were made with the saline only group. E) Quantification of the metastatic cells in lung sections using the deep learning method. *p < 0.05, **p < 0.01, ***p < 0.001, ****p < 0.0001; Student's *t*‐test. Data represent the mean ± SEM.

To monitor tumor metastasis in vivo, 4T1 cells were infected with a plasmid encoding firefly luciferase Luc2P. Tumor metastasis was visualized in vivo and ex vivo with an IVIS Spectrum imaging system. In vivo images were captured weekly (Figure S5A, Supporting Information). In the first 5 weeks, the growth of the primary tumors was the main signal observed. Starting in the 6th week, luciferase signals from tumor metastases were observed. The strong signals from the primary tumors limited the detection of the weaker signals from the tumor metastases. To address this problem, we shielded the primary tumors with black paper, which resulted in better detection of luciferase signals from the metastases. Strong thoracic luciferase signals associated with metastases were observed in the saline only and PTX groups, while the PG3, NP^+^, and PTX‐loaded NP^+^ treatment groups exhibited weak thoracic luciferase signals (**Figure** **5**A). Total photon flux indicated significantly less tumor metastasis in the PTX‐loaded NP^+^ group than in either the PTX or PTX‐loaded NP^−^ groups (Figure 5B). Because 4T1 cells preferentially create metastases in the lungs, mouse lungs were collected for ex vivo imaging immediately after the mice were sacrificed (Figure 5C).^[^ ^19^ ^]^ Tumor metastasis was greater in the saline only, PTX, and NP^−^ groups than in the PTX‐loaded NP^+^ group based on imaging and quantification of photon flux, indicating that the G3‐C12_5_‐DEEA_20_ NPs inhibited PTX‐promoted metastasis.

Metastatic nodules on the lung lobes were visible to the naked eye and in the images (Figure 5D, Figure S5B,C, Supporting Information). A higher number of metastatic nodules with larger sizes were observed in the saline only, PTX, and PTX‐loaded NP^−^ groups. Based on the total area of metastatic nodules on the lobes, significantly less metastasis was observed in the PTX‐loaded NP^+^ treatment group. Metastatic nodules were observed more clearly in H&E‐stained sections (Figure S5D, Supporting Information). More metastatic nodules that were larger in size were observed in the saline only, PTX, and PTX‐loaded NP^−^ groups than in the PTX‐loaded NP^+^ treatment group. The areas of the metastatic nodules were quantified using a deep learning method with 98% training accuracy and 91% validation accuracy (Figure 5E, Figure S5E–H, Supporting Information).^[^ ^20^ ^]^ A probability map was used to identify metastatic cells, and the results of quantitation showed that PTX‐loaded NPs^+^ significantly inhibited lung metastasis. Tumor metastasis in lung tissues was also observed via Ki‐67 staining for cell proliferation (Figure S5I, Supporting Information). Strong fluorescence from metastatic nodules was observed in lung sections from the saline only and PTX groups, whereas the PTX‐loaded NP^+^ group exhibited a background signal similar to that of healthy lung tissues.

### 2.7 Paclitaxel‐Loaded G3‐C12_5_‐DEEA_20_ Nanoparticles Reduce Cell‐Free Nucleic Acid and Inflammatory Cytokine Levels in Mouse Serum

**Figure 6:**
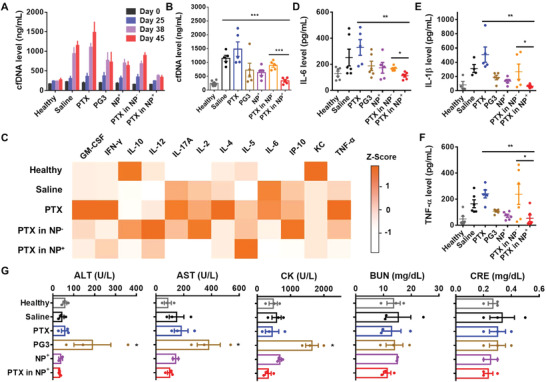
Inflammatory cytokine and biochemical analyses of mouse serum. A) Time‐dependent cfDNA levels in the serum of BALB/c mice bearing 4T1 tumors. B) Cell‐free DNA levels in mouse serum immediately after the mice were sacrificed. C) Inflammatory cytokine levels (Z scores) in mouse serum detected with IsoPlexis chips. Only detectable cytokines are shown. D–F) IL‐6, IL‐1*β*, and TNF‐*α* levels in mouse serum detected by ELISA. G) Serum biochemical tests showing the levels of ALT, AST, CK, BUN, and CRE. Comparisons were made with the healthy group. *p < 0.05, **p < 0.01, ***p < 0.001, ****p < 0.0001; Student's *t*‐test. Data represent the mean ± SD.

Elevated cfNA levels in the systemic circulation are positively correlated with the progression of tumor metastasis in mice. cfDNA levels in BALB/c mouse serum were monitored every two weeks and showed a close correlation with the degree of tumor metastasis (**Figure** **6**A). In the first 3–4 weeks, cfDNA levels were steady as tumor metastasis had not yet occurred. Starting at week 5, prior to observation of metastasis, the cfDNA levels started to increase. Starting at week 6, tumor metastasis was observed, and cfDNA levels continued to increase. The mice were sacrificed when the tumor size exceeded 1000 mm^3^; at this point, the average cfDNA levels in the saline only and PTX groups were much higher than that in the healthy group (Figure 6B). In contrast, the PTX‐loaded NP^+^ group exhibited a cfDNA level that was similar to that in healthy mice and significantly lower than that in the PTX‐loaded NP^−^ group. These results demonstrated the cfNA scavenging ability of the G3‐C12_5_‐DEEA_20_ NPs.

Tumor metastasis can be promoted by activating TLR‐related pathways and inducing inflammatory cytokine secretion.^[^ ^9a^ ^]^ Cytokine levels in mouse serum were analyzed with IsoPlexis High‐Plex chips. 16 cytokines were assayed, and their levels were converted into standard scores (Figure 6C, Figure S6A, Supporting Information). Serum from the PTX treatment group showed the highest levels of inflammatory cytokines, followed by the PTX‐loaded NP^−^ and saline only groups. The PTX‐loaded NP^+^ group showed low cytokine levels, similar to those in the healthy group. The levels of IFN‐*γ*, IL‐6, and TNF‐*α* (TLR‐related cytokines) were elevated by PTX treatment, as were the levels of other inflammatory cytokines—GM‐CSF, IL‐17A, IL‐2, IL‐4, and IP‐10. Treatment with PTX‐loaded NPs^+^ reduced the levels of most of these cytokines in the systemic circulation. The levels of IL‐6, IL‐1*β*, and TNF‐*α* in serum were also tested by ELISA (Figure 6D–F). The PTX‐loaded NP^+^ group exhibited significantly lower cytokine levels than the PTX and PTX‐loaded NP^−^ groups. Together, these results indicated that using cationic G3‐C12_5_‐DEEA_20_ NPs to deliver PTX reduced the inflammatory cytokine levels induced by PTX treatment.

### 2.8 G3‐C12_5_‐DEEA_20_ Nanoparticles Exhibit No Significant Observable Side Effects In Vivo

The body weights of mice treated with the various nanomaterials did not decrease significantly during the in vivo study (Figure S6B, Supporting Information). However, several mice treated with PAMAM‐G3 died before the primary tumors reached a volume of 1000 mm^3^, indicating the toxicity of PAMAM‐G3 during this treatment course (Figure 4B). Biochemical tests of serum collected from sacrificed mice were used to assess major organ function (Figure 6G). Serum from the PAMAM‐G3 group exhibited much higher AST and ALT levels than serum from the healthy group, indicating toxicity to the liver. High levels of CK revealed potential muscle toxicity. In contrast, treatment with G3‐C12_5_‐DEEA_20_ NPs did not result in evident toxicity to these organs. No significant change was detected in BUN and CRE levels, suggesting little toxicity to the kidney. H&E analysis also showed liver toxicity induced by PAMAM‐G3 treatment, while treatment with the G3‐C12_5_‐DEEA_20_ NPs showed little toxicity (Figure S6C, Supporting Information). No significant tissue damage was observed in other organs.

### 2.9 Cationic Nanoparticles Inhibit Paclitaxel‐Induced Human Breast Cancer Metastasis in NSG Mice

**Figure 7:**
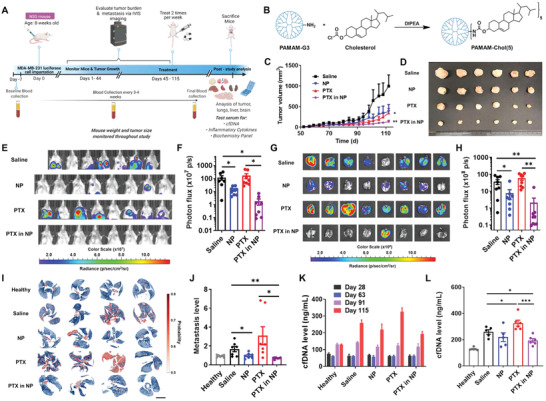
Effect of PTX‐loaded NPs in a human breast cancer NSG mouse model. A) Treatment timeline for the human breast cancer metastasis model. Schematic created with BioRender.com. B) Structure and synthetic route of PAMAM‐Chol(5). C,D) Primary tumor growth curves and tumor photos of MDA‐MB‐231 human breast cancer‐bearing NSG mice treated with different therapeutic agents. Polymers (15 mg kg^−1^) and PTX (4.5 mg kg^−1^) were administered via i.p. injection two times a week. Mice were sacrificed after the tumor volumes exceeded 1000 mm^3^. E) IVIS images of mice on day 114. Primary tumors were covered with black paper to shield their bright signals to better detect metastases. F) Total photon flux from the IVIS images. G) Ex vivo images of lungs showing tumor metastasis signals collected immediately after the mice were sacrificed. H) Total photon flux from the ex vivo images. I) Probability maps generated by a deep‐learning method that identifies metastatic tissue. The probability heatmap indicates the probability that each cell is metastatic. Scale bar, 6 mm. J) Quantification of the metastatic cells in lung sections using the deep learning method. Metastasis levels were normalized to the healthy group. K) Time‐dependent cfDNA levels in the serum of NSG mice bearing MDA‐MB‐231 tumors. L) Cell‐free DNA levels in mouse serum immediately after the mice were sacrificed. *p < 0.05, **p < 0.01, ***p < 0.001, ****p < 0.0001; Student's *t*‐test. Data represent the mean ± SEM.

To expand the antimetastatic effect of the cationic NPs to a human cancer model, an MDA‐MB‐231 human breast cancer NSG mouse model was constructed. MDA‐MB‐231 cells were transfected with the plasmid encoding firefly luciferase Luc2P and injected subcutaneously into the mammary glands of female NSG mice (**Figure** **7**A). Cholesterol‐modified PAMAM‐G3 NPs with a similar size (125.0 ± 10.5 nm) and zeta potential (56.4 ± 1.1 mV) were used in this model (Figure 7B, Figure S7A–D, Supporting Information). Treatments were administered via i.p. injection, and included saline only, PTX, NPs, and PTX‐loaded NPs. Again, PTX inhibited primary tumor growth in this model, while PTX‐loaded NPs inhibited primary tumor growth more efficiently (Figure 7C,D, Figure S7E,F, Supporting Information). Lung metastasis was observed by detecting the bioluminescence generated from the Luc2P‐transfected cancer cells. Both in vivo and ex vivo imaging showed that more metastasis occurred in the saline‐ and PTX‐treated groups, while less metastasis was observed in the NP‐ and PTX‐loaded NP groups (Figure 7E–H). The deep learning method was also applied to this model. The probability map identified the metastatic cells in lung sections, and the quantitative results showed that PTX‐loaded NPs significantly inhibited lung metastasis (Figure 7I,J, Figure S7G, Supporting Information). The cfDNA levels in serum increased as tumor metastasis were generated. When the mice were sacrificed, the PTX treatment group showed a significant higher cfDNA level than the saline group, while the PTX‐loaded NP treatment group showed a significant lower level (Figure 7K,L). In addition, no body weight loss or organ damage was observed in any of the NP treatment groups (Figure S7H,I, Supporting Information). Cholesterol‐modified PAMAM‐G3 NPs used in this model had a similar PAMAM‐based structure with NP^+^, but modified with cholesterol instead of dodecyl chain. The promising performance of these two cationic NPs in two mouse models further confirmed their anti‐metastasis effect, and suggested that the effect is not limited to a specific structure but has the potential to be applied to a broader landscape.

## 3 Discussion

PTX, one of the most widely used chemotherapeutic agents for breast cancer, exhibits primary tumor growth inhibition with high efficacy but is a double‐edged sword, as it also induces tumor metastasis.^[^ ^21^ ^]^ Different mechanisms of PTX‐induced metastasis have been reported, but few therapies have been developed to mitigate this effect. Here, we show that PTX can induce breast cancer metastasis via increasing circulating cfNAs and activation of TLR signaling pathways. A therapeutic nanocarrier‐based strategy was developed to inhibit PTX‐induced metastasis by scavenging cfNAs.

Chemotherapeutics, including PTX, have been reported to induce the production of DAMPs during the treatment of primary tumors.^[^ ^22^ ^]^ DAMPs are endogenous molecules released from dying cells, and cfNAs are a main type of DAMP induced by chemotherapy. In our study, PTX DAMP media—cancer cell growth media collected from MDA‐MB‐231 cells treated with PTX with a negligible amount of PTX remaining—exhibited higher levels of cfDNA and RNA than control media collected from untreated cells. In previous studies, cfNAs in PTX DAMP media have been shown to activate TLRs on cancer cells and immune cells and induce the production of inflammatory cytokines.^[^ ^23^ ^]^ In our study, PTX DAMP media induced the activation of TLRs 3, 8, and 9 to a similar level as standard nucleic acid TLR agonists, whereas the control media did not have this effect. PTX DAMP media also increased the expression of TLR pathway‐related genes and the levels of the inflammatory cytokines TNF‐*α*, IL‐6, and IL‐1*β* released by immune cells. Moreover, PTX DAMP‐induced inflammation promoted the migration and invasion of cancer cells, as demonstrated in scratch wound healing and transwell assays. PTX‐induced metastasis was also observed in a 4T1 mammary cancer‐bearing mouse model. CfDNA levels in mouse serum were higher in the PTX treatment group than in the saline only group. The cfDNA levels continued to increase as the primary tumors grew and as metastasis increased. Higher levels of inflammatory cytokines were also detected in the serum of the PTX group by ELISA and with IsoPlexis High‐Plex automated chips. Lung metastases observed using several different techniques indicated that PTX can induce breast cancer metastasis.

The ability of PAMAM‐G3 polymers to scavenge cfNAs has been demonstrated previously.^[^ ^14a^ ^]^ However, unmodified hydrophilic PAMAM‐G3 cannot be loaded with chemotherapeutics, and PAMAM has shown toxicity that may preclude its long‐term treatment applications. Therefore, we designed a series of polymers based on PAMAM dendrimers to fabricate therapeutic nanocarriers with reduced side effects. G3‐C12_5_‐DEEA_20_ was chosen as the chemotherapeutic nanocarrier because of its high DNA binding ability and TLR inhibition activity, low toxicity, and smaller nanoparticle (NP) size.

The cationic G3‐C12_5_‐DEEA_20_ NPs were found to bind cfNAs both extracellularly and intracellularly. These NPs inhibited CpG ODN‐induced TLR9 activation even when the NPs were added 2 h before the agonist and were removed when the agonist was added, indicating that they acted intracellularly. Trapping cfNAs inside cells may help reduce their concentration in the tumor microenvironment, thus reducing inflammation. The cationic NPs also promoted the cellular uptake and retention of extracellular cfNAs, preventing their spread into the circulation. CLSM revealed the colocalization of CpG ODNs and cationic NPs with lysosomes, indicating that the NPs bound with cfDNA and trapped it in lysosomes.

The ability of the G3‐C12_5_‐DEEA_20_ NPs to inhibit PTX‐induced metastasis was studied in vitro and in vivo. In vitro, the NPs reduced cfNA levels in PTX DAMP media, inhibited TLR activation, and reduced the expression of TLR‐related genes and the levels of inflammatory TLR‐related cytokines. The NPs also inhibited cancer cell migration and invasion in scratch wound healing and transwell experiments. In the in vivo studies, 4T1 cells were chosen because they are highly aggressive murine mammary carcinoma cells with great potential to induce metastasis. Firefly luciferase genes were transfected into 4T1 cells to monitor the time and location of metastasis formation without the need to sacrifice the mice. Initially, a relatively low number of 4T1 cells were inoculated to prevent the primary tumors from growing too fast, which would affect metastasis observation. Additionally, a relatively low dosage of PTX was administered to allow for metastasis. Nevertheless, even with this setup of a relatively low tumor burden and a low drug dosage, primary tumor growth inhibition was still greater in the PTX treatment group than in the untreated or NP only group. However, free PTX even induced more metastasis than saline alone, whereas groups treated with cationic nanomaterials (polymer or NPs) exhibited less metastasis. Interestingly, PTX‐loaded anionic NPs, which lacked the ability to scavenge cfNAs, inhibited primary tumor growth but not tumor metastasis, confirming that the cationic NPs inhibited tumor metastasis via cfNA scavenging. This is consistent with previous findings in a murine model of pancreatic cancer.^[^ ^14a^ ^]^ It is tempting to speculate that by simply reducing primary tumor growth in the PTX‐loaded cationic NP treatment group, we were able to reduce metastatic spread. However, the PTX and PTX‐loaded anionic NP treatments generated more metastasis while inhibiting primary tumor growth. Metastatic spread was inhibited in the cationic polymer and NP treatment groups, although these mice had primary tumors comparable in size to those in the saline only group. These results provide evidence that even in the presence of a sizable primary tumor, metastatic spread can be reduced when mice are treated with cationic polymers or NPs.

Deep learning methods have been developed for medical imaging diagnosis and classification, such for the identification of cancer metastasis in whole slide images (WSIs). However, there have not been many applications of deep learning to evaluate the therapeutic efficacy of cancer treatments, such as dynamically assessing cancer progression and cancer cell migration in vivo. Here, we adopted a previously published deep learning model called the “neural conditional random field”^[^ ^24^ ^]^ to distinguish and quantify metastatic cancerous regions in mouse lungs. The deep learning architecture and training datasets are described in Section 5. Accuracy values of 98% and 91% were achieved during training and validation, respectively, indicating that the deep learning‐based quantification method provided a robust, accurate, and objective approach to evaluate breast cancer metastasis in the lungs of mice.

Increased levels of cfNAs in serum are only one of several factors that could promote breast cancer metastasis. This study demonstrates that cationic PAMAM‐based NPs can bind cfNAs, thereby providing a method to target one of the DAMPs that promote metastasis. Most proinflammatory DAMPs are negatively charged, but further mechanistic studies are required to determine whether these cationic NPs also bind other types of DAMPs or circulating molecules that promote metastasis. In addition, it is necessary to investigate the NPs in different in vivo cancer models. Surgically resection of primary tumors together with chemotherapy administration in an animal model would better mimic clinical conditions. The human breast cancer cell line MDA‐MB‐231 is a good candidate for the construction of a new mouse model. More models should be included to evaluate therapeutic nanocarriers in future investigations before considering clinical trials.

## 4 Conclusion

Therapeutic nanocarriers were developed to deliver chemotherapeutics while simultaneously inhibiting chemotherapy‐induced breast cancer metastasis. By encapsulating a chemotherapeutic in a cationic nanocarrier, inhibition of tumor progression at both the primary and metastatic sites was achieved. This therapeutic nanocarrier strategy improves the therapeutic effects of chemotherapy, reduces the risk of metastasis, and should also be applicable to other cancer models.

## 5 Experimental Section

### Materials and Reagents

1‐Dodecanol, *N*,*N*‐dimethylethanolamine (DMEA), *N*,*N*‐diethylethanolamine (DEEA), and *N*,*N*‐dibutylethanolamine (DBEA) were purchased from Aladdin (China). 1,1’‐Carbonyldiimidazole (CDI) was purchased from Energy Chemical (China). PAMAM dendrimers generations 2.5, 3, and 4 were purchased from Dendritech, Inc. (USA). 1‐Dodecylamine and PTX were purchased from Sigma–Aldrich (USA). 1‐Ethyl‐3‐(3‐dimethylaminopropyl)carbodiimide (EDC), *N*‐hydroxysuccinimide (NHS), and solvents for HPLC were purchased from Thermo Fisher (USA). Sephadex LH‐20 was purchased from Pharmacia (USA). DMSO for polymer synthesis was dried over CaH_2_ and distilled under reduced pressure prior to use. Methanol was dried with molecular sieves. Ultrapure water (18.2 MΩ) was used in this study. Other solvents used for polymer synthesis were purchased from Sinopharm Chemical Reagent (China).

### Synthetic Routes

To obtain G3‐C12_5_‐DEEA_20_, CDI (1.62 g, 10 mmol) was dissolved in 30 mL of dichloromethane (DCM). DEEA (0.586 g, 5 mmol) dissolved in 5 mL of DCM was then added dropwise to the CDI solution with stirring, and the reaction was stirred at room temperature for 24 h. The reaction mixture was washed three times with saturated saline, and the organic phase was collected and dried over anhydrous MgSO_4_. After filtration and removal of solvent under reduced pressure, a pale yellow oily product (DEEA‐CDI) was obtained in a yield of 76%. The chemical structure of the product was characterized with a Bruker Avance III 400 NMR instrument. ^1^H NMR (TMS, CDCl_3_, ppm): 8.15 (s, 1H, —NCHN—), 7.48 (s, 1H, —OCONCH_2_CH_2_N—), 7.02 (s, 1H, —OCONCH_2_CH_2_N—), 4.48 (t, 2H, —OCH_2_CH_2_N—), 2.80 (t, 2H, —OCH_2_CH_2_N—), 1.25–1.50 (m, 4H, —N(CH_2_CH_3_)_2_), 0.93 (t, 6H, —N(CH_2_CH_3_)_2_). DMEA‐CDI, DBEA‐CDI, and DDC‐CDI were synthesized using similar methods except DMEA, DBEA, and DDC were used as the starting alcohols, respectively. In the next step, PAMAM‐G3 (30 mg, 4.34 µmol) was dissolved in 2 mL of dry DMSO, and DDC‐CDI (6.1 mg, 21.7 µmol) was added. The mixture was stirred at 40 °C overnight. Then, DEEA‐CDI (118.0 mg, 555.52 µmol) was added, and the mixture was stirred at 40 °C for another 24 h. The product was purified with a Sephadex LH‐20 column with methanol as the eluent. After removal of the solvent by rotary evaporation, the product was obtained in a yield of 89%. ^1^H NMR (TMS, CDCl3, ppm): 4.15 (—NHCOOCH_2_CH_2_N—), 4.05 (—NHCOOCH_2_CH_2_CH_2_—), 3.50–2.20 (—OCH_2_CH_2_N—, —N(CH_2_CH_3_)_2_, and all other protons from PAMAM), 1.60 (—NHCOOCH_2_CH_2_CH_2_—), 1.25 (—NHCOOCH_2_(CH_2_)_10_CH_3_), 1.05 (—N(CH_2_CH_3_)_2_), 0.95 (—NHCOOCH_2_(CH_2_)_10_CH_3_). Other PAMAM derivatives were synthesized using similar procedures with different amounts of DMEA‐CDI, DEEA‐CDI, DBEA‐CDI, and DDC‐CDI. To obtain G2.5‐C12_5_, PAMAM‐G2.5 (63 mg, 10 µmol) was dissolved in 5 mL of DMSO. EDC (48 mg, 250 µmol) and NHS (12 mg, 100 µmol) were added to the solution, which was stirred for 30 min. Then, 1‐dodecylamine (9.3 mg, 50 µmol) was added, and the mixture was stirred at room temperature overnight and then dialyzed against water for 72 h. The mixture was lyophilized to obtain the product in a yield of 94%.

### Fabrication of Paclitaxel‐Loaded G3‐C125‐DEEA20 Nanoparticles

300 µg of PTX was dissolved in 200 µL of chloroform with 1 mg of G3‐C12_5_‐DEEA_20_. 2 mL of water was added, and the mixture was sonicated for 2 min. An additional 2 mL of water was added, and then the solution was concentrated to 1 mL by rotary evaporation. Unloaded PTX was removed by centrifugation at 3000 rpm for 30 min due to its low solubility in water. The hydrodynamic diameter and zeta potential of the NPs were measured with a Malvern Nano ZS90 Zetasizer. Drug loading efficiency was determined by HPLC (Agilent 1260 infinity, USA) using a reversed‐phase HC‐C18(2) column (4.6×150 mm, pore size 4 µm, Agilent, USA). A water solution of NPs (1 mg mL^−1^) was mixed with an equal volume of DCM under vigorous vortexing. The water phase was discarded, and the DCM was removed under vacuum. The solid was redissolved in a 50/50 (v/v) mixture of acetonitrile and water for HPLC analysis. The mobile phase consisted of a 38/31/31 (v/v/v) mixture of acetonitrile, methanol and water at a flow rate of 1 mL min^−1^. The PTX peak at 227 nm was detected with a UV–vis HPLC detector. Drug release profiles were determined by HPLC using the same conditions described above. Briefly, 1 mg of NPs was dispersed in a mixture of 0.1 m citric acid and 0.2 m Na_2_HPO_4_ buffer solutions with 0.1% w/v Tween 80 (pH = 7.4 or 5.5).^[^ ^25^ ^]^ The suspension was placed in a cellulose dialysis bag (Spectra/Por 3, MWCO 3500, Spectrum, USA). The dialysis bag was immersed in 10 mL of release medium in a centrifuge tube, and the tube was shaken on a shaking bed. After different time intervals, the dialysis bag was transferred to a new centrifuge tube with fresh release media. The collected samples (1 mL) were extracted with equal volumes of DCM. Then, the DCM was removed, and the samples were redissolved in 1 mL of mobile phase for HPLC analysis.

### Cell Lines and Animals

The human breast cancer cell line MDA‐MB‐231, the murine leukemia virus‐transformed macrophage cell line RAW 264.7, the human monocyte cell line THP‐1, and the mouse breast cancer cell line 4T1 were purchased from American Type Culture Collection (ATCC). Human TLR‐expressing HEK‐Blue hTLR3, hTLR8, and hTLR9 cells were purchased from Invitrogen. Human kidney 293T cells were purchased from GenHunter. All cell lines were cultured at 37 °C in a humidified atmosphere with 5% CO_2_. MDA‐MB‐231, HEK‐Blue hTLR, and RAW 264.7 cells were cultured in DMEM; THP‐1 and 4T1 cells were cultured in RPMI 1640 supplemented with 10% fetal bovine serum (FBS), penicillin (100 U mL^−1^), and streptomycin (100 µg mL^−1^). The medium for HEK‐Blue hTLR cells was further supplemented with Normocin (100 µg mL^−1^), blasticidin (100 µg mL^−1^), and zeocin (100 µg mL^−1^). The medium for THP‐1 cells was supplemented with 2‐mercaptoethanol (0.05 mm). All animal experiments were approved by Columbia University's Institutional Animal Care and Use Committee (IACUC) (AC‐AABC8557). Female BALB/c mice (5 weeks old) were purchased from Jackson Labs. The mice were housed under pathogen‐free conditions in the animal facility (Institute of Comparative Medicine) at Columbia University. Mice were housed in groups of four per cage and were acclimatized to the animal facility environment for 1 week prior to the initiation of the experimental procedures.

### Structural Optimization of Cell‐Free Nucleic Acid Scavengers

The cytotoxicity of the nanomaterials was measured using a Cell Counting Kit‐8 (CCK‐8) assay (Dojindo, USA). MDA‐MB‐231 and HEK‐TLR9 cells were plated in 96‐well plates at 1 × 10^4^ cells per well, and aliquots of NPs at various concentrations were added. After 72 h of incubation, 20 µL of CCK‐8 reagent was added to each well for incubation at 37 °C for 1 h. The absorbance of each well was measured at 450 nm with a FLUOstar Optima FL microplate reader. Absorbance readings were normalized to that of untreated MDA‐MB‐231 cells. The DNA binding ability of the NPs was assessed using a Quant‐iT PicoGreen DNA Assay Kit (Fisher Scientific, USA). Concentrated PicoGreen solutions were diluted 2000‐fold in TE buffer (Fisher Scientific, USA). Salmon sperm DNA (Fisher Scientific, USA) was added as a standard to reach a final concentration of 2 µg mL^−1^. The mixture was incubated at 37 °C for 30 min. Different NPs (50 µL well^−1^) were then added to a 96‐well black plate. The mixture of PicoGreen and salmon sperm DNA (50 µL well^−1^) was added to the same plate, which was shaken for 30 min. Fluorescence (Ex/Em: 490 nm/520 nm) was measured using a microplate reader. The ability of the NPs to inhibit TLR activation was assessed using HEK‐Blue hTLR3/8/9 cells. An appropriate number of cells per well for each cell line (5 × 10^4^ for TLR3, 4 × 10^4^ for TLR8, and 8 × 10^4^ for TLR9) was seeded into 96‐well plates in DMEM. The following agonist concentrations were used to activate HEK‐Blue hTLR3, hTLR8, and hTLR9 cells, respectively: 1 µg mL^−1^ poly I:C dsRNA analog, 500 ng mL^−1^ ORN06/LyoVec, and 1 µg mL^−1^ ODN BW006. Agonists were added to the cells alone or together with the library of 14 soluble PAMAM NPs (2 µg mL^−1^). The cells were incubated for 24 h, and 50 µL of the supernatant media was transferred to a new 96‐well plate containing 150 µL of QUANTI‐Blue solution. The absorbance of the secreted alkaline phosphatase released from the HEK‐Blue hTLR cells was measured using a microplate reader at 620 nm, and the readings were normalized to the average absorbance of the untreated cells.

### Cellular Uptake and Retention

For the cellular uptake study, MDA‐MB‐231 cells were seeded at 6 × 10^4^ cells per well in 24‐well plates and cultured overnight. Then, the cells were incubated with media containing CpG‐Cy5 (5 µg mL^−1^) and cationic NPs (10 µg mL^−1^) for different lengths of time. The cells were washed three times with PBS and lysed. The amount of CpG ODN taken up by the cells was determined by measuring the fluorescence of Cy5 with a microplate reader. The total amount of protein was quantified with a BCA kit. CpG was labeled with Cy5‐NHS (Fisher Scientific, USA) in a mass ratio of 50:1 by stirring overnight at 4 °C.

For the retention study, after the cells were incubated as described above for 12 h, the medium was aspirated, and the cells were washed three times with PBS. After the cells were cultured in fresh media for an additional 2 to 6 h, the cells were collected and lysed to measure the Cy5 fluorescence and total protein content. For the intracellular localization study, circular 12‐mm diameter glass coverslips (Fisher Scientific, USA) were washed with 70% ethanol solution and then flame sterilized before being placed in a 24‐well plate. To promote cellular adherence, 500 µL of 0.1% gelatin solution (Millipore, USA) was placed on the coverslips. After leaving at room temperature for 1 h, the gelatin solution was removed and the coverslips were washed with PBS and allowed to air dry for 15 min. MDA‐MB‐231 cells were seeded at 4 × 10^4^ cells per well on the coverslips in the 24‐well plate and allowed to adhere overnight. The cells were then divided into three groups and treated in duplicate as follows: untreated MDA‐MB‐231 media, 1 µg mL^−1^ CpG‐Cy5 (IDT, USA), or 1 µg mL^−1^ CpG‐Cy5 + 2 µg mL^−1^ FITC‐labeled NPs. After incubation for 6 h, the media from all groups was removed and replaced with basal MDA‐MB‐231 media containing 50 nM LysoTracker Red DND‐99 (Invitrogen, USA) for 30 min to stain lysosomes. The cells were then washed three times with PBS, fixed with 4% paraformaldehyde for 20 min, washed another three times with PBS, and stained with DAPI for 10 min. The coverslips were mounted onto microscope slides (Fisher Scientific, USA) using Fluoromount‐G (Southern Biotech, USA) and imaged with a Confocal Nikon Ti Eclipse inverted microscope. NPs were labeled with FITC‐NHS (Fisher Scientific, USA) in a mass ratio of 50:1 by stirring overnight at 4 °C.

### Preparation and Characterization of Paclitaxel Damage‐Associated Molecular Pattern Media

PTX powder was diluted in DMSO to obtain a 250 µM stock solution. MDA‐MB‐231 cells were plated at 1.8 × 10^6^ cells per dish in 10‐cm dishes and cultured overnight. To prepare PTX DAMP media, the existing media was replaced with 10 mL of MDA‐MB‐231 cells supplemented with 10 µL of PTX stock solution to obtain a final concentration of 250 nM PTX. The same volume of culture media plus 10 µL of DMSO was used to create control media. After 6 h of incubation, the media was aspirated and replaced by DMEM, and the cells were incubated for an additional 72 h. The media was then harvested and used immediately or stored at −80 °C until use. To determine an appropriate concentration of PTX to generate PTX DAMP media, it was key that cell death was achieved while a negligible amount of PTX remained in the media. A low level of residual PTX in the media was necessary to avoid toxicity in subsequent cell‐based assays. An assay was performed to measure the amount of residual PTX remaining after the cells were treated. MDA‐MB‐231 cells were plated at 1 × 10^4^ cells per well in 96‐well plates and treated with different concentrations of PTX labeled with Oregon Green (Invitrogen, USA) for 6 h. Then, the media was replaced with DMEM, and the plates were incubated for 72 h. 50 µL of the supernatant was transferred to a black 96‐well plate. 50 µL of DMSO was added to each well to ensure solubility, and the fluorescence signal (Ex/Em: 490 nm/520 nm) was measured with a microplate reader. A standard curve was generated by using different concentrations of Oregon Green‐labeled PTX. cfDNA levels in DAMP media were measured with a Quant‐iT PicoGreen DNA Assay Kit (Fisher Scientific, USA). DAMP medium (50 µL well^−1^) was mixed with PicoGreen solutions (diluted 1000‐fold in TE buffer, 50 µL well^−1^) in a 96‐well black plate. A standard curve was constructed with lambda DNA (Fisher Scientific, USA). The plate was shaken for 30 min, and the fluorescence (Ex/Em: 490 nm/520 nm) was measured with a microplate reader. cfRNA levels were measured similarly, except that DAMP media (10 µL well^−1^) was assayed using a Quant‐iT RNA Assay Kit (diluted 200‐fold in TE buffer, 100 µL well^−1^, Fisher Scientific, USA) in a 96‐well black plate. TLR activation and cell viability tests were performed using similar methods.

### Quantitative PCR and ELISA

THP‐1 cells (1 × 10^6^ cells per well) were cultured in 6‐well plates overnight. DAMP media (1 mL) was mixed with culture media (1 mL) and added to each well. After incubation for 24, 48, and 72 h, the medium was collected for ELISA, and the cells were washed three times with PBS. Total RNA was isolated with a Direct‐zol RNA Miniprep kit (Zymo Research, USA), and the concentration of the isolated RNA was measured with a Denovix DS‐11+ spectrophotometer. Equal amounts of RNA in different samples were reverse transcribed into cDNA with an iScript cDNA Synthesis Kit (Bio‐Rad, USA). cDNA was amplified with SsoAdvanced Universal SYBR Green Supermix (Bio‐Rad, USA) using a QuantStudio 3 Real‐Time PCR System. The primer sequences are shown in Table S3, Supporting Information. Media collected from THP‐1 or RAW 264.7 cells was centrifuged to remove cells and debris. The levels of TNF‐*α*, IL‐1*β*, and IL‐6 were measured using ELISA kits (Invitrogen, USA).

### Cell Migration and Invasion Assays

Cell migration was evaluated using a scratch wound healing assay. MDA‐MB‐231 cells (1.5 × 10^5^ cells per well) were cultured in a 24‐well plate overnight. Wounds were created by using a 200‐µL plastic pipette tip on the cell monolayer. The media were replaced with DAMP media with or without 20 µg mL^−1^ cationic NPs, and the cells were cultured for an additional 48 h. Images of the scratch wounds were captured at 0, 24, and 48 h with a Nikon Eclipse TE 2000‐U microscope. Wound closure was measured with ImageJ. Cell invasion was evaluated with a transwell assay. BioCoat Matrigel invasion chambers with BD Matrigel matrix (Corning, USA) were rehydrated. MDA‐MB‐231 cells (5 × 10^5^) were seeded onto the top chambers in 700 µL of DMEM with 10% heat‐inactivated FBS, and 500 µL of the same media was placed in the bottom chambers. Cells were allowed to adhere for 4 h, and the media in the top and bottom chambers were aspirated and replaced with media appropriate for each different treatment group. In the control group, only DMEM was added. In the treatment groups, PTX DAMP media was added to the top and bottom chambers either alone or with 25 µg mL^−1^ NPs. The chambers were incubated at 37 °C for 24 h and then removed, and the cells in the top Matrigel layer were withdrawn with a damp cotton swab. Chambers were immersed once in PBS to wash and were then placed in wells containing 4% paraformaldehyde (Fisher Scientific, USA) for 20 min in the dark to fix the cells on the basolateral surface. Next, the chambers were dipped twice in PBS to wash and then placed in 1% crystal violet solution (Sigma–Aldrich, USA) for 15 min to stain the cells. Residual crystal violet was removed from the chambers by sequential washes with water, and the chambers were left at room temperature to dry overnight. Cells that invaded the bottom of the Matrigel chambers were imaged and quantified using ImageJ.

### Luciferase Labeling of 4T1 Cells

To follow the growth and metastasis of cancer cells in vivo, 4T1 cells were infected with a plasmid encoding firefly luciferase Luc2P. 293T cells were cultured to 70% confluence in a T25 flask and transfected using JetPRIME (Polyplus, USA). Briefly, DNA solution containing 0.8 g of pRSV‐Rev (Addgene #12253), 1.0 g of pVSVG (Addgene #12259), 1.2 g of pMDLg/pRRE (Addgene #12251), and 1 g of pHIV‐Luc‐ZsGreen (Addgene # 39196) was prepared. DNA was diluted in 200 µL of jetPRIME buffer at a ratio of 2:1 JetPRIME reagent/DNA. After incubation at room temperature for 10 min, transfection mix was added dropwise to the 293T cells. 4 h after transfection, the medium was exchanged for fresh medium containing 4 mm caffeine (Sigma–Aldrich, USA). The supernatant was collected 48 h after transfection and filtered through 0.45‐micron filters. Polybrene (Sigma–Aldrich, USA) was added at 1000×. 4T1 cells were cultured to ≈90% confluence, and the media was replaced with viral supernatant followed by centrifugation for 2 h at 2400 rpm. Cells were incubated overnight, and fresh media was added the following morning. Following inoculation, cells were sorted to obtain a population of pure viable ZsGreen‐positive cells. Cells were trypsinized to form a single‐cell solution and stained with DAPI (Life Technologies, USA). Flow cytometry analysis was performed to sort for GFP positivity using a BD FACSAria cell sorter (BD Biosciences, USA). Forward scatter area versus forward scatter height profiles were used to eliminate cell doublets. Dead cells were eliminated by excluding DAPI^+^ cells. Each cell population underwent two consecutive rounds of purification to achieve a final purity of >99%.

### Breast Cancer Metastasis Mouse Model

4T1 cells were labeled with a pHIV‐luc‐zsgreen vector to stably express luciferase. Luciferase‐expressing 4T1 cells (2 × 10^5^) were injected subcutaneously into the fourth mammary glands of BALB/c female mice. 6 mice without tumor cell injection were regarded as the healthy control group. Tumor‐bearing animals were randomized into 6 groups (*n* = 6 per group): Saline, PTX (4.5 mg kg^−1^), PAMAM‐G3 (15 mg kg^−1^), NP^+^ (15 mg kg^−1^), PTX in NP^+^ (PTX 4.5 mg kg^−1^, NP^+^ 15 mg kg^−1^), and PTX in NP^−^ (PTX 4.5 mg kg^−1^, NP^−^ 15 mg kg^−1^). PTX solutions were diluted in 10% Cremophor EL (Sigma–Aldrich, USA), 10% ethanol, and 80% saline solution. Treatment was administered via i.p. injection three times per week. Once the tumors reached a palpable size (≈50 mm^3^), tumor growth was recorded every other day with digital calipers by the same person throughout the study for consistency. Tumor volumes were calculated using the formula 0.5 × length (mm) × width^2^ (mm). The mice were euthanized when the primary tumor volumes reached 1000 mm^3^. Kaplan–Meier survival curves were plotted. The body weights of the mice were measured every other day. Blood was collected from the submandibular vein every 2 weeks. Blood samples were centrifuged at 4000 rpm for 10 min, and the supernatant was collected as serum samples to measure cfNA levels. The tumors, blood, and main organs were collected for further analysis when the mice were euthanized, and a final serum sample was obtained for ELISAs and biochemical tests. Cytokine levels in serum were analyzed with a High‐Plex chip (IsoPlexis, USA). The mouse adaptive immune panel was used for the detection of 16 murine inflammatory cytokines: RANTES, IL‐6, IL‐17A, MCP‐1, IL‐4, MIP‐1a, IL‐12, IP‐10, IFN‐*γ*, IL‐2, GM‐CSF, IL‐5, TNF‐*α*, IL‐1*β*, and IL‐10.

### Immunofluorescence and Immunohistochemical Staining

Primary tumor, heart, lung, liver, spleen, and kidney tissues were fixed in 4% paraformaldehyde overnight and embedded in paraffin to obtain 5‐µm thick sections. All tissues were stained with hematoxylin and eosin (H&E) to visualize tumor progression, metastasis, and side effects. Primary tumor tissues were stained with TUNEL (Click‐iT Plus TUNEL Assay Kit, Alexa Fluor 594 dye, Invitrogen, USA) and Ki‐67 antibody (1:500, Novus Biologicals, catalog no. NB11089717F) to visualize tumor cell apoptosis and proliferation. Immunofluorescence images were acquired with an Axio Observer 7 inverted microscope (Zeiss, USA). Immunohistochemistry images were acquired with an Aperio Digital Pathology Slide Scanner (Leica, USA).

### Deep Learning

The deep learning architecture combined a convolutional neural network (CNN) module for extracting image features and a conditional random field (CRF) module for improving margin prediction. The CNN architecture used was the residual network (ResNet‐18), based on the idea that each layer learns residual structures with reference to input layers instead of unreferenced structures. ResNet‐18 consists of 18 layers and 5 convolutional stages. First, the input was given a convolutional layer with a 7 × 7 kernel size and stride of 2. Then, both a batch normalization layer and a ReLU activation function were added after each convolutional layer. A skip connection linked the output of a residual block (3 × 3, 64 filters) to the next. The output of this residual block set was added to the output of two convolution layers (3 × 3 kernel and 128 filters). A similar process was performed until the fourth and final residual set (512 filters) was achieved. Global average pooling was applied to the output of the final residual block, and the output feature map was applied to the fully connected layers, followed by a SoftMax function to receive the final output as a vector of probabilities for the identified classes. CRF was the statistical modeling method used for pattern recognition. It took into account the spatial correlations of smaller image patches partitioned from the WSIs during the training and estimated the marginal label distribution of those patches through the minimization of KL divergence between the CRF distribution *P*(*Y*) and a simpler distribution *Q*(*Y*), where *Y* denotes the random variable of image patch labels. The Camelyon16 datasets were used as the training datasets, which are WSIs of sentinel lymph nodes with labels annotated by pathologists on the tumor area. 10 000 tumor image patches and 6000 normal image patches sampled from 10 gigapixel images as training sets and 8000 tumor images and 6000 normal patches sampled from 10 gigapixel images as validation sets were generated. Training reached 98% accuracy, and 91% validation accuracy was achieved. Mouse lung WSIs were preprocessed to obtain a binary mask of tissues by using the Otsu algorithm, and the trained model was applied to level 0 of the WSI, which was obtained at 40× magnification. The probability maps of the metastatic tumor lesions in the WSI were able to be learned by the model. The pixel number was summarized with a threshold of 0.6 on the probability map. To predict the levels of metastasis, the probability map was filtered for the positive values and calculated the mean value. All of the quantification processes were written in Python using PyTorch, and the WSIs were processed with OpenSlide, which is a C library used to load the WSIs at different magnification levels.

### IVIS Imaging

The luciferase signals from the 4T1 cells were monitored via in vivo imaging every week to evaluate tumor progression in the primary and metastatic sites. Luciferin solutions (150 mg kg^−1^ body weight) were administered to the mice via i.p. injection, and bioluminescence was detected immediately using an IVIS Lumina III system (PerkinElmer, USA). When the mice were euthanized at the end of the study, the main organs (lungs, liver, kidneys, heart, and spleen) were collected for ex vivo imaging with the IVIS system. The bioluminescence intensity was analyzed using Living Image software (Caliper Life Sciences, USA). To evaluate the biodistribution of the polymers, 4T1 cells (2 × 10^6^) were subcutaneously injected into the mammary glands of BALB/c female mice. After the tumor volumes reached 200 mm^3^, 15 mg kg^−1^ Cy5‐labeled PAMAM‐G3, NPs^+^ or NPs^−^ were administered via i.p. injection (*n* = 3 per group). The mice were sacrificed after 24 h, and the main organs were collected. Then, ex vivo imaging was performed immediately using the IVIS system to detect the fluorescence of Cy5 (Ex/Em: 630/680). Fluorescence intensity was analyzed using Living Image software. NPs were labeled with Cy5‐NHS (Fisher Scientific, USA) in a mass ratio of 50:1 by stirring overnight at 4 °C.

### Statistical Analysis

Statistical analysis was performed using GraphPad Prism 7. Experimental results are displayed as the mean ± SD or mean ± SEM. Differences between two groups were tested with an unpaired two‐tailed Student's *t*‐test. Differences between survival curves were analyzed using a log‐rank test.

## Conflict of Interest

The authors declare no conflict of interest.

## Supporting information

Supporting Information

## Data Availability

The data that support the findings of this study are available in the supplementary material of this article.

## References

[1] R. L. Siegel , K. D. Miller , A. Jemal , Ca‐Cancer J. Clin. 2020, 70, 7. 31912902 10.3322/caac.21590

[2] F. Bray , J. Ferlay , I. Soerjomataram , R. L. Siegel , L. A. Torre , A. Jemal , Ca‐Cancer J. Clin. 2018, 68, 394. 30207593 10.3322/caac.21492

[3] a) N. Harbeck , F. Penault‐Llorca , J. Cortes , M. Gnant , N. Houssami , P. Poortmans , K. Ruddy , J. Tsang , F. Cardoso , Nat. Rev. Dis. Primers 2019, 5, 66; 31548545 10.1038/s41572-019-0111-2

[4] a) G. S. Karagiannis , J. M. Pastoriza , Y. Wang , A. S. Harney , D. Entenberg , J. Pignatelli , V. P. Sharma , E. A. Xue , E. Cheng , T. M. D'Alfonso , J. G. Jones , J. Anampa , T. E. Rohan , J. A. Sparano , J. S. Condeelis , M. H. Oktay , Sci. Transl. Med. 2017, 9, eaan0026; 28724576 10.1126/scitranslmed.aao3817

[5] a) D. Panigrahy , A. Gartung , J. Yang , H. Yang , M. M. Gilligan , M. L. Sulciner , S. S. Bhasin , D. R. Bielenberg , J. Chang , B. A. Schmidt , J. Piwowarski , A. Fishbein , D. Soler‐Ferran , M. A. Sparks , S. J. Staffa , V. Sukhatme , B. D. Hammock , M. W. Kieran , S. Huang , M. Bhasin , C. N. Serhan , V. P. Sukhatme , J. Clin. Invest. 2019, 129, 2964; 31205032 10.1172/JCI127282 PMC6597207

[6] a) S. Wu , K. M. Turner , N. Nguyen , R. Raviram , M. Erb , J. Santini , J. Luebeck , U. Rajkumar , Y. Diao , B. Li , W. Zhang , N. Jameson , M. R. Corces , J. M. Granja , X. Chen , C. Coruh , A. Abnousi , J. Houston , Z. Ye , R. Hu , M. Yu , H. Kim , J. A. Law , R. G. W. Verhaak , M. Hu , F. B. Furnari , H. Y. Chang , B. Ren , V. Bafna , P. S. Mischel , Nature 2019, 575, 699; 31748743 10.1038/s41586-019-1763-5 PMC7094777

[7] a) J. Tuomela , J. Sandholm , M. Kaakinen , A. Patel , J. H. Kauppila , J. Ilvesaro , D. Chen , K. W. Harris , D. Graves , K. S. Selander , Breast Cancer Res. Treat. 2013, 142, 477; 24212717 10.1007/s10549-013-2762-0 PMC4238912

[8] a) L. C. Kidd , E. N. Rogers , S. T. Yeyeodu , D. Z. Jones , K. S. Kimbro , Breast Cancer 2013, 5, 43; 24648757 10.2147/BCTT.S29172 PMC3929246

[9] a) J. P. Pradere , D. H. Dapito , R. F. Schwabe , Oncogene 2014, 33, 3485; 23934186 10.1038/onc.2013.302 PMC4059777

[10] H. Schwarzenbach , D. S. Hoon , K. Pantel , Nat. Rev. Cancer 2011, 11, 426. 21562580 10.1038/nrc3066

[11] a) A. J. Bronkhorst , V. Ungerer , S. Holdenrieder , Biomol. Detect. Quantif. 2019, 17, 100087; 30923679 10.1016/j.bdq.2019.100087 PMC6425120

[12] J. Lee , J. W. Sohn , Y. Zhang , K. W. Leong , D. Pisetsky , B. A. Sullenger , Proc. Natl. Acad. Sci. U. S. A. 2011, 108, 14055. 21844380 10.1073/pnas.1105777108 PMC3161575

[13] a) H. Liang , B. Peng , C. Dong , L. Liu , J. Mao , S. Wei , X. Wang , H. Xu , J. Shen , H. Q. Mao , X. Gao , K. W. Leong , Y. Chen , Nat. Commun. 2018, 9, 4291; 30327464 10.1038/s41467-018-06603-5 PMC6191420

[14] a) I. Naqvi , R. Gunaratne , J. E. McDade , A. Moreno , R. E. Rempel , D. C. Rouse , S. G. Herrera , D. S. Pisetsky , J. Lee , R. R. White , B. A. Sullenger , Mol. Ther. 2018, 26, 1020; 29550075 10.1016/j.ymthe.2018.02.018 PMC6079560

[15] a) D. Peer , J. M. Karp , S. Hong , O. C. Farokhzad , R. Margalit , R. Langer , Nat. Nanotechnol. 2007, 2, 751; 18654426 10.1038/nnano.2007.387

[16] a) X. Lu , L. Miao , W. Gao , Z. Chen , K. J. McHugh , Y. Sun , Z. Tochka , S. Tomasic , K. Sadtler , A. Hyacinthe , Y. Huang , T. Graf , Q. Hu , M. Sarmadi , R. Langer , D. G. Anderson , A. Jaklenec , Sci. Transl. Med. 2020, 12, eaaz6606; 32801144 10.1126/scitranslmed.aaz6606 PMC9019818

[17] L. C. Bailey‐Downs , J. E. Thorpe , B. C. Disch , A. Bastian , P. J. Hauser , T. Farasyn , W. L. Berry , R. E. Hurst , M. A. Ihnat , PLoS One 2014, 9, e98624. 24878664 10.1371/journal.pone.0098624 PMC4039511

[18] J. Kota , R. R. Chivukula , K. A. O'Donnell , E. A. Wentzel , C. L. Montgomery , H. W. Hwang , T. C. Chang , P. Vivekanandan , M. Torbenson , K. R. Clark , J. R. Mendell , J. T. Mendell , Cell 2009, 137, 1005. 19524505 10.1016/j.cell.2009.04.021 PMC2722880

[19] M. Yousefi , R. Nosrati , A. Salmaninejad , S. Dehghani , A. Shahryari , A. Saberi , Cell. Oncol. 2018, 41, 123. 10.1007/s13402-018-0376-6 PMC12995240 29568985

[20] B. E. Bejnordi , M. Veta , P. J. van Diest , B. van Ginneken , N. Karssemeijer , G. Litjens , J. van der Laak , C. C. The , M. Hermsen , Q. F. Manson , M. Balkenhol , O. Geessink , N. Stathonikos , M. C. van Dijk , P. Bult , F. Beca , A. H. Beck , D. Wang , A. Khosla , R. Gargeya , H. Irshad , A. Zhong , Q. Dou , Q. Li , H. Chen , H. J. Lin , P. A. Heng , C. Hass , E. Bruni , Q. Wong , et al., JAMA, J. Am. Med. Assoc. 2017, 318, 2199.

[21] a) J. D. Middleton , D. G. Stover , T. Hai , Int. J. Mol. Sci. 2018, 19, 3333; 30373101 10.3390/ijms19113333 PMC6274941

[22] a) M. J. M. Magbanua , L. B. Swigart , H. T. Wu , G. L. Hirst , C. Yau , D. M. Wolf , A. Tin , R. Salari , S. Shchegrova , H. Pawar , A. L. Delson , A. DeMichele , M. C. Liu , A. J. Chien , D. Tripathy , S. Asare , C. J. Lin , P. Billings , A. Aleshin , H. Sethi , M. Louie , B. Zimmermann , L. J. Esserman , L. J. van ’t Veer , Ann. Oncol. 2021, 32, 229; 33232761 10.1016/j.annonc.2020.11.007 PMC9348585

[23] a) G. S. Karagiannis , J. S. Condeelis , M. H. Oktay , Cancer Res. 2019, 79, 4567; 31431464 10.1158/0008-5472.CAN-19-1147 PMC6744993

[24] Y. Li , W. Ping , arXiv:1806.07064, 2018, https://arxiv.org/abs/1806.07064

[25] P. D. Ramirez‐Garcia , J. S. Retamal , P. Shenoy , W. Imlach , M. Sykes , N. Truong , L. Constandil , T. Pelissier , C. J. Nowell , S. Y. Khor , L. M. Layani , C. Lumb , D. P. Poole , T. Lieu , G. D. Stewart , Q. N. Mai , D. D. Jensen , R. Latorre , N. N. Scheff , B. L. Schmidt , J. F. Quinn , M. R. Whittaker , N. A. Veldhuis , T. P. Davis , N. W. Bunnett , Nat. Nanotechnol. 2019, 14, 1150. 31686009 10.1038/s41565-019-0568-x PMC7765343

