# Supplementary material for: Therapeutic Nanocarriers Inhibit Chemotherapy‐Induced Breast Cancer Metastasis
Source: Adv Sci (Weinh). 2022 Oct 11;9(33):2203949. doi: 10.1002/advs.202203949 (PMC9685442; doi:10.1002/advs.202203949)
Supplement: Supplementary file 1 — Supporting Information [file ADVS-9-2203949-s001.pdf]

ADVANCED SCIENCE
Open Access

Supporting Information

for *Adv. Sci.*, DOI 10.1002/advs.202203949

Therapeutic Nanocarriers Inhibit Chemotherapy-Induced Breast Cancer Metastasis

*Tianyu Li, Tolulope Akinade, Jie Zhou, Hongxia Wang, Qisong Tong, Siyu He, Emily Rinebold, Luis E. Valencia Salazar, Divya Bhansali, Yiling Zhong, Jing Ruan, Jinzhi Du, Piero Dalerba and Kam W. Leong**

Supporting Information

Therapeutic Nanocarriers to Inhibit Chemotherapy-Induced Breast Cancer Metastasis

*Tianyu Li, Tolulope Akinade, Jie Zhou, Hongxia Wang, Qisong Tong, Siyu He, Emily Rinebold, Luis E. Valencia Salazar, Divya Bhansali, Yiling Zhong, Jing Ruan, Jinzhi Du, Piero Dalerba, Kam W. Leong****

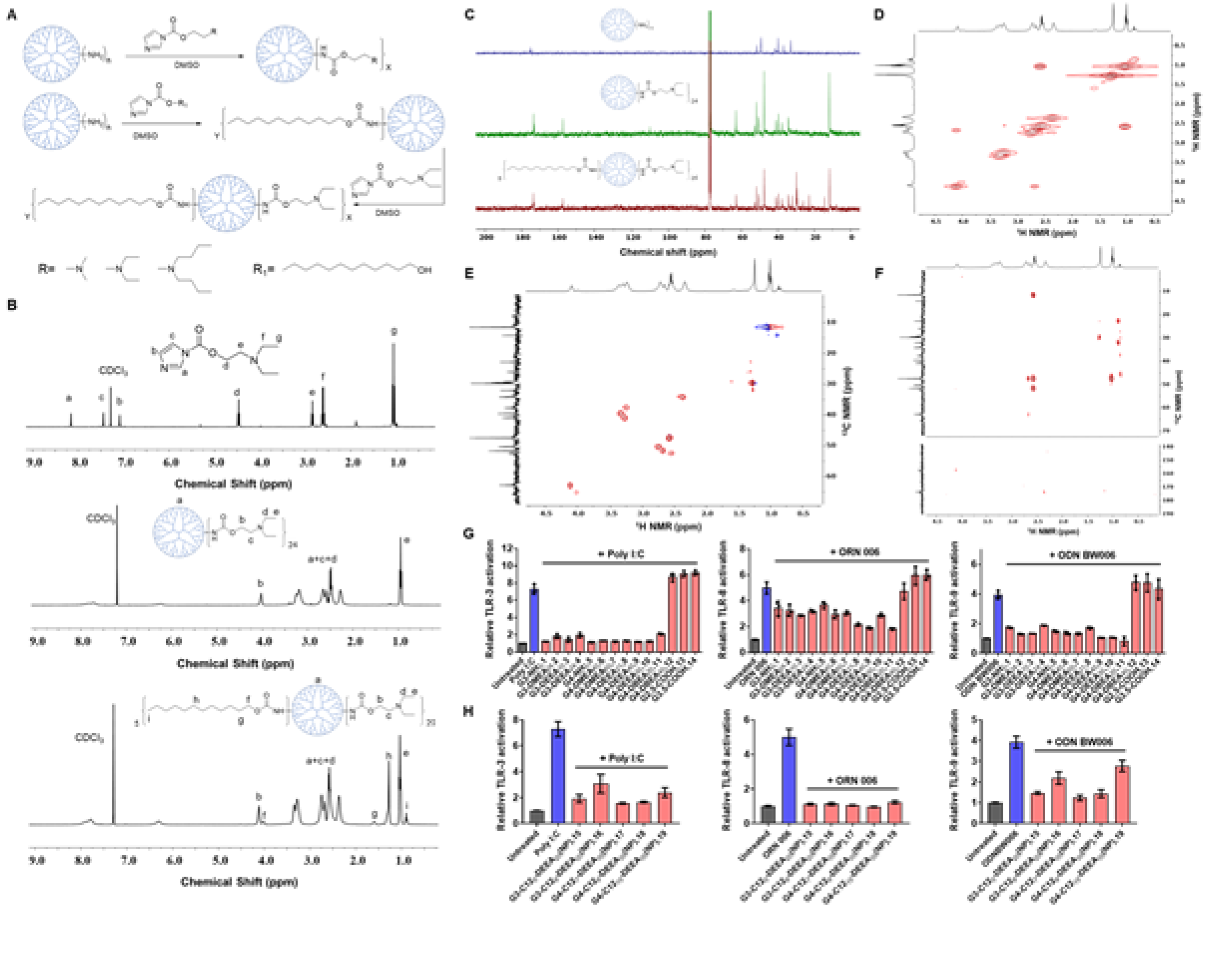

Fig. S1. Structures and characterization of PAMAM dendrimer derivatives. (A) Synthesis route of PAMAM dendrimer derivatives. (B) ^{1}H-NMR spectra of DEEA-CDI, G3-DEEA$_{24}$, and G3-C12$_{5}$-DEEA$_{20}$. (C) ^{13}C-NMR spectra of PAMAM-G3, G3-DEEA$_{24}$, and G3-C12$_{5}$-

DEEA$_{20}$. (D-F) ^{1}H-^{1}H COSY, ^{1}H-^{13}C HSQC and ^{1}H-^{13}C HMBC spectra of G3-C12$_{5}$-DEEA$_{20}$. (G) TLR3, 8, and 9 inhibition efficiency of PAMAM dendrimer derivatives. (H) TLR3, 8, and 9 inhibition efficiency of nanoparticles based on PAMAM dendrimers. Data were normalized to the untreated group and indicate mean ± SD.

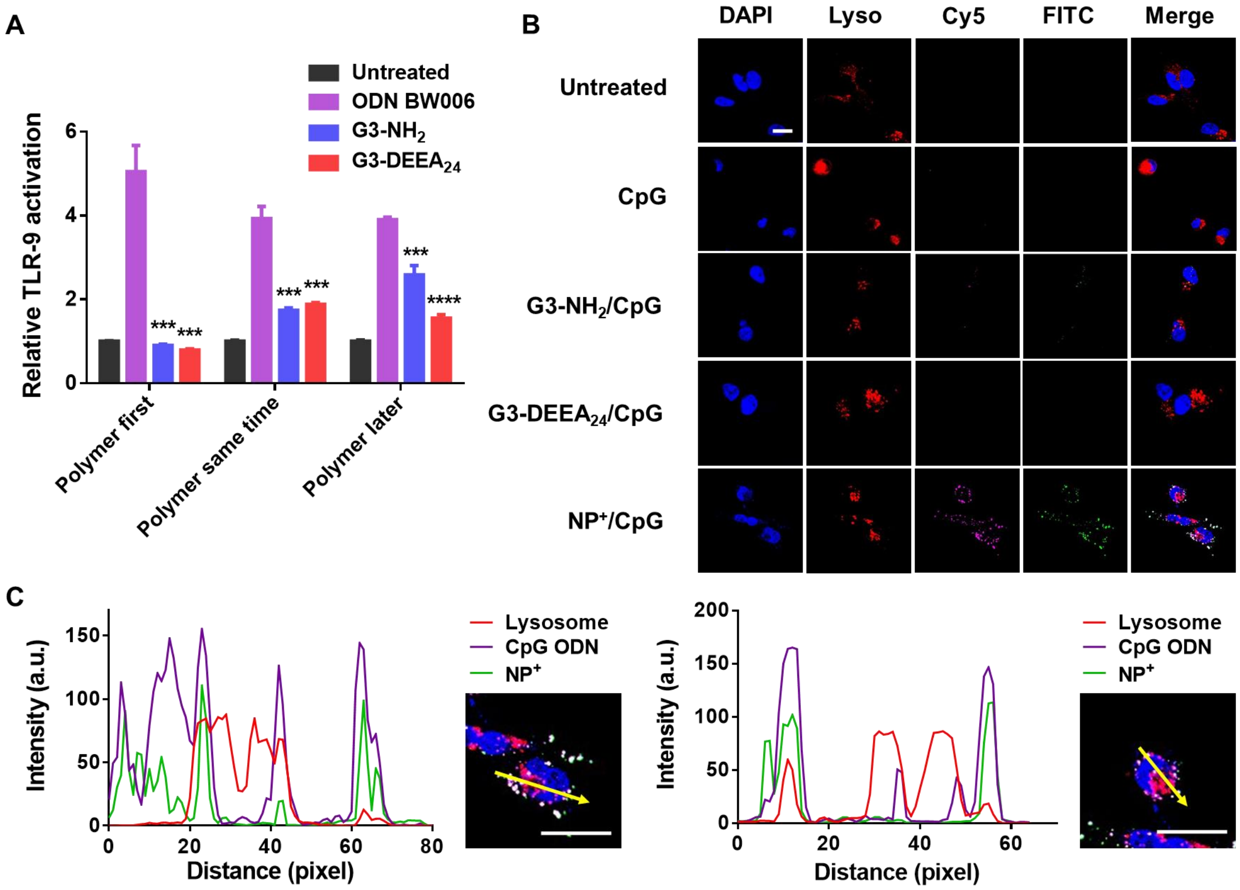

Fig. S2. TLR inhibition and intracellular trafficking of nanoparticles. (A) TLR9 activation assay in which cationic nanoparticles were added at different times. Cationic nanomaterials were added 2 h before, 2 h after, or at the same time as agonist treatment. Data were normalized to the untreated group. Comparisons are with the agonist-only group. *P<0.05, **P<0.01, ***P<0.001, ****P<0.0001; Student's t-test. Data represent mean ± SD. (B) Fluorescent images showing cell uptake of CpG ODNs and cationic nanoparticles. (C) Colocalization profiles along the yellow arrow in cells treated with NP^+ and CpG. MDA-MB-231 cells were treated for 2 h. Cell nuclei were stained with DAPI, lysosomes with LysoTracker Red, CpG ODNs with Cy5, and cationic nanomaterials with FITC. Colocalization profiles of CpG ODN, NP^+, and lysosomes along the direction of the arrow. Scale bar, 20 μm.

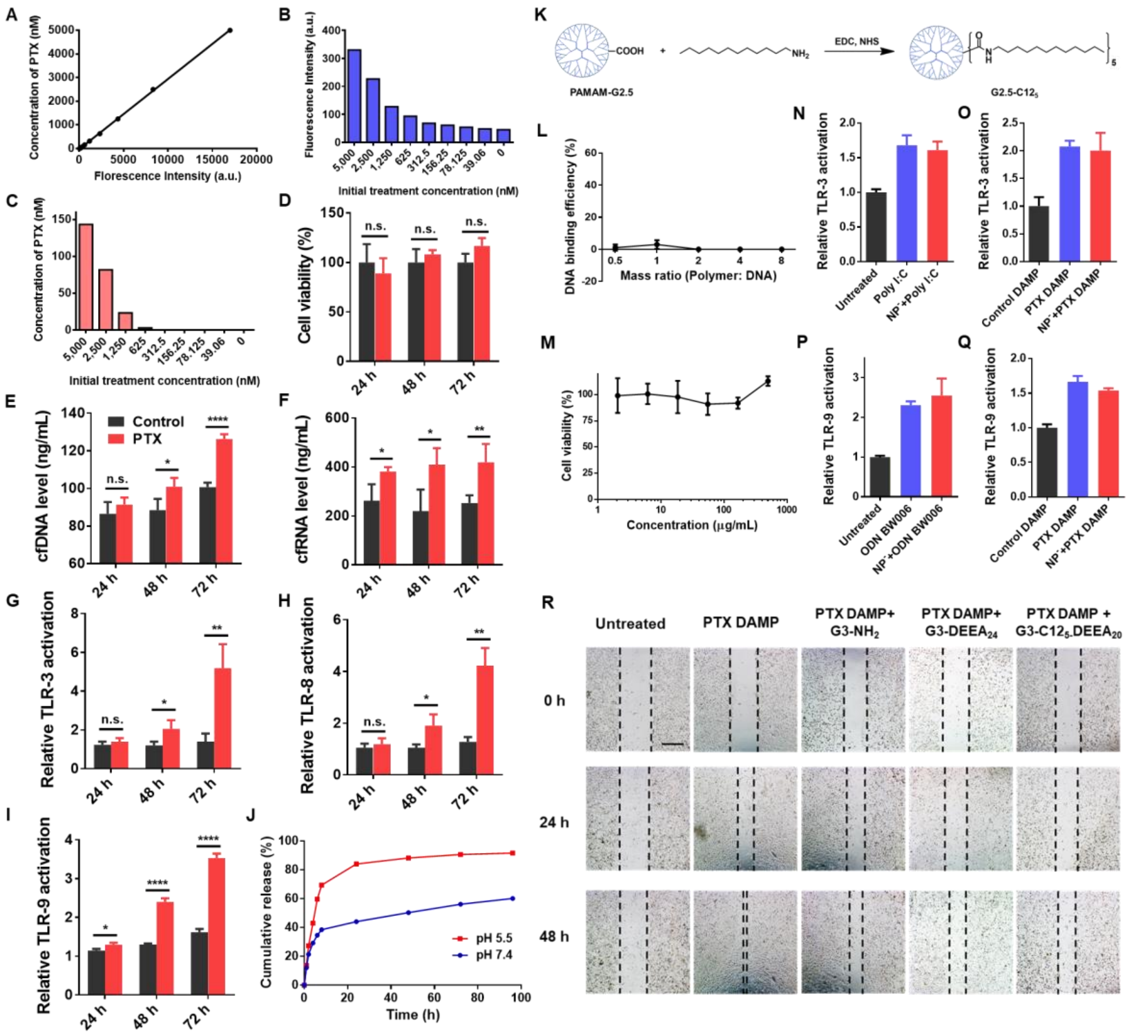

Fig. S3. Nanoparticle inhibition of paclitaxel/DAMP-induced cancer cell migration and invasion. (A) Standard curve in media containing varying levels of Oregon Green 488-labeled PTX. (B) Fluorescence intensity of Oregon Green 488-labeled PTX remaining in PTX DAMP media prepared with different initial PTX concentrations. (C) Concentrations of PTX remaining in PTX DAMP solutions. (D) Cell viability of MDA-MB-231 cells treated with PTX DAMP or control DAMP media. (E, F) Cell-free DNA and RNA levels in PTX DAMP and control DAMP. (G-I) TLR3, 8, and 9 activations by PTX DAMP and control DAMP. **P*<0.05, ***P*<0.01, ****P*<0.001, *****P*<0.0001; Student's *t*-test. Data represent mean ± SD. (J) Release profiles of PTX loaded in NP^{+}. PTX-loaded NP^{+} were dialyzed in citric acid-sodium dihydrogen phosphate buffer at pH 5.5 and pH 7.4 at 37 °C. Released PTX amounts were measured using HPLC. (K) Structure and synthesis route of NP^{-}. (L) DNA binding efficiency of NP^{-} at different polymer:DNA ratios. (M) Cell viability test using NP^{-} in MDA-MB-231 cells for 72 h. (N, O) NP^{-} did not inhibit TLR3 activation caused by poly I:C or PTX DAMP. (P, Q) NP^{-} did not inhibit TLR9 activation caused by ODN BW006 or PTX DAMP. Data were normalized to the untreated and control DAMP groups. (R) Scratch wound healing images showing migration of MDA-MB-231 cells. Scale bar, 500 μm.

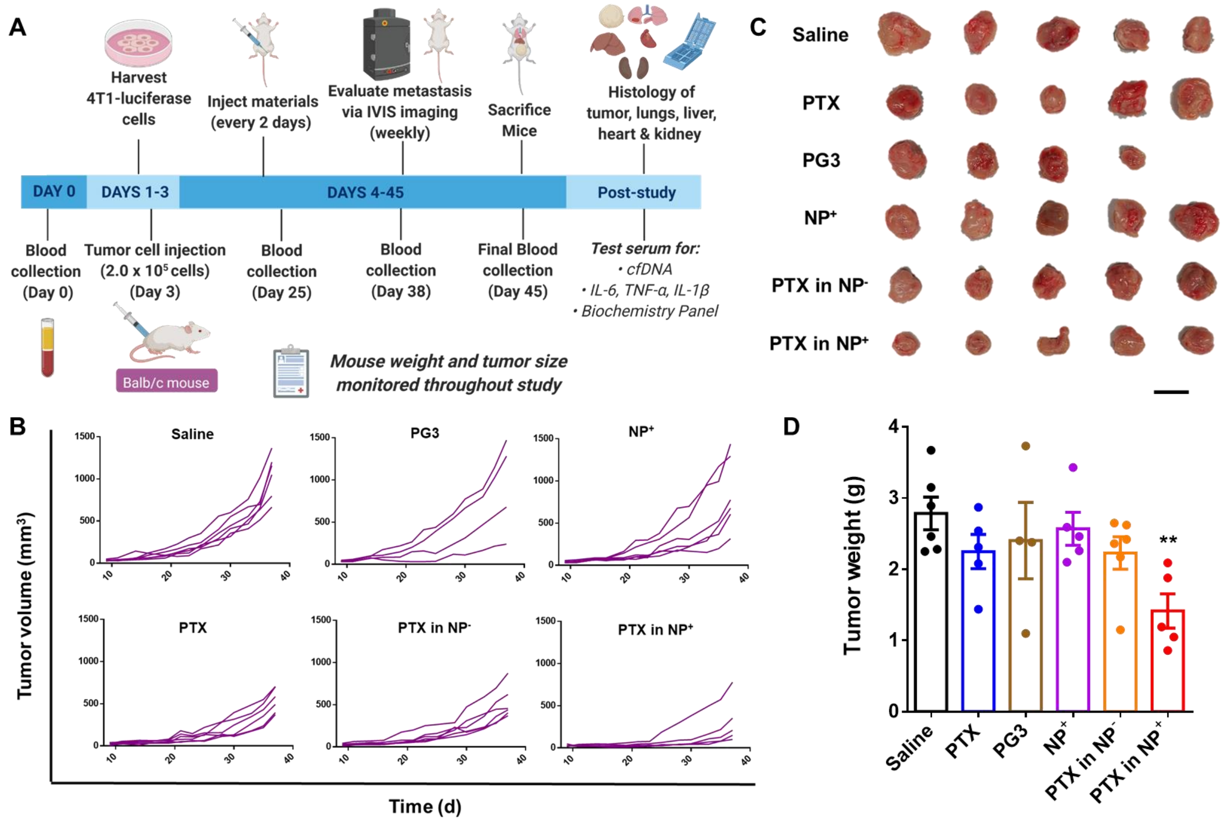

Fig. S4. Effect of PTX-loaded NPs on primary tumor progression. (A) Treatment timeline for the mouse breast cancer metastasis model. Schematic created with BioRender.com. (B) Tumor growth curves for individual mice. (C) Tumor images. Scale bar, 2 cm. (D) Tumor weights. Comparisons are with the saline-only group. *P<0.05, **P<0.01, ***P<0.001, ****P<0.0001; Student's t-test. Data represent mean ± SEM.

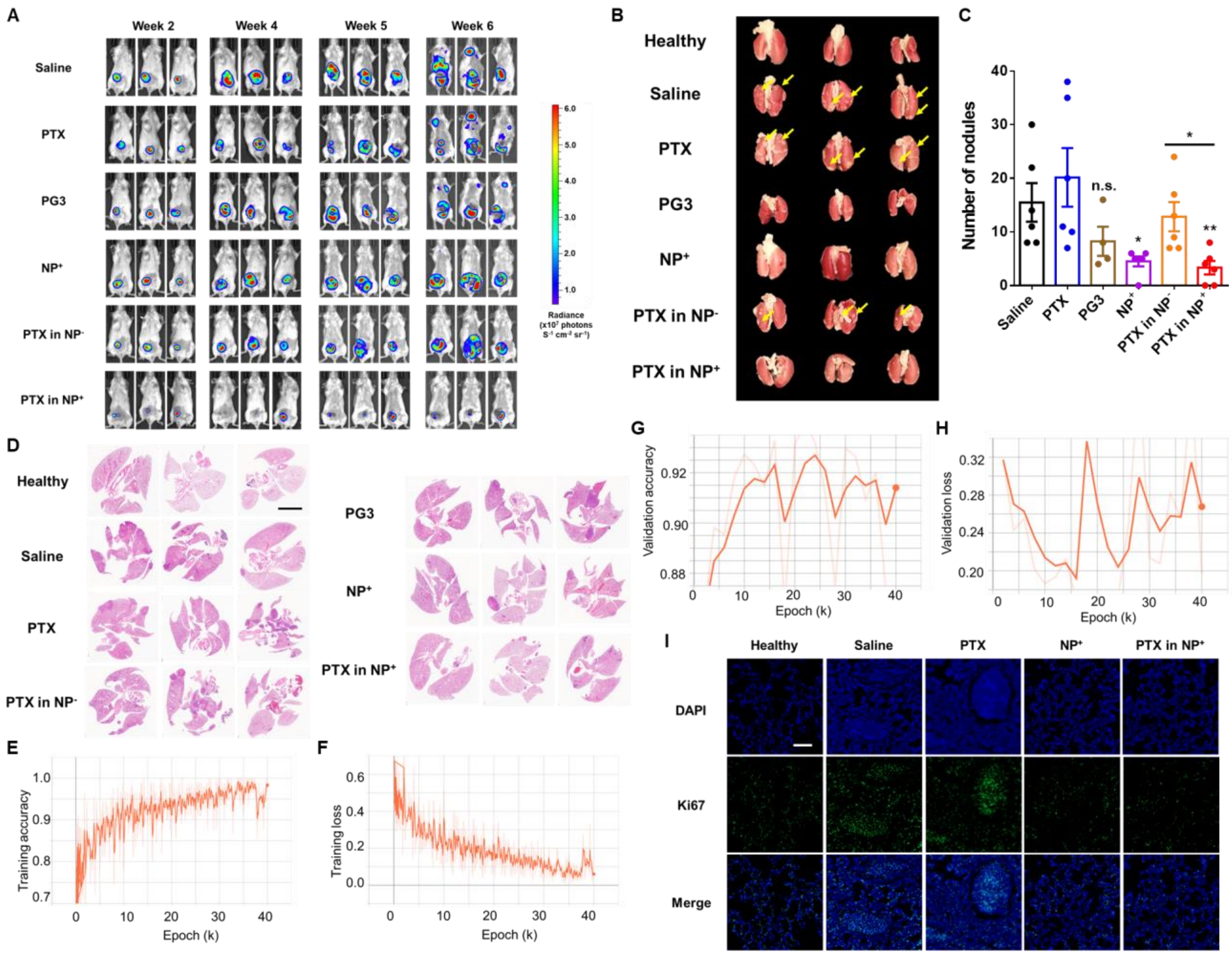

Fig. S5. Effect of PTX-loaded cationic NPs on tumor metastasis. (A) *In vivo* imaging showing 4T1-luciferase tumor growth on both primary and metastatic sites. (B) Lung photos exhibiting visible metastasis nodules. (C) Quantification of visible nodules on lung lobes. Comparisons are with the saline-only group. **P*<0.05, ***P*<0.01, ****P*<0.001, *****P*<0.0001; Student's *t*-test. Data represent mean ± SEM. (D) Lung sections with H&E staining showing metastasis nodules. Scale bar, 2 mm. (E, F) Accuracy and loss over training with deep learning method. (G, H) Accuracy and loss over validation with deep learning method (k = 10^3). (I) Ki67 staining of lung tissues showing metastasis nodules. Scale bar, 50 µm.

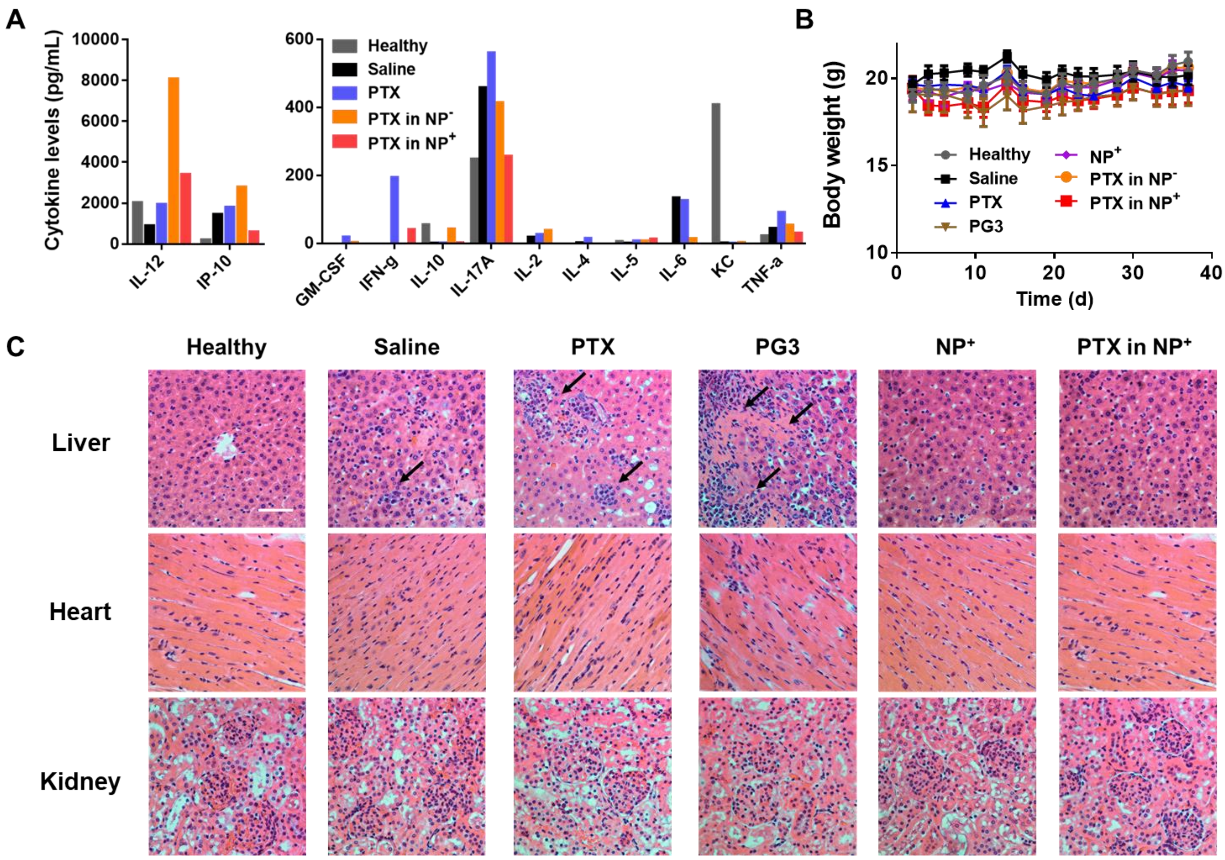

Fig. S6. Inflammatory cytokine levels in mouse serum and evaluation of side effects. (A) Inflammatory cytokine levels in mouse serum at the final time point detected with IsoPlexis chips. (B) Body weights of healthy and 4T1 mammary tumor-bearing BALB/c mice in different treatment groups. (C) H&E staining images of livers, hearts and kidneys. Arrows indicate metastatic cancer cells and necrosis areas in liver tissues. Scale bar, 50 μm.

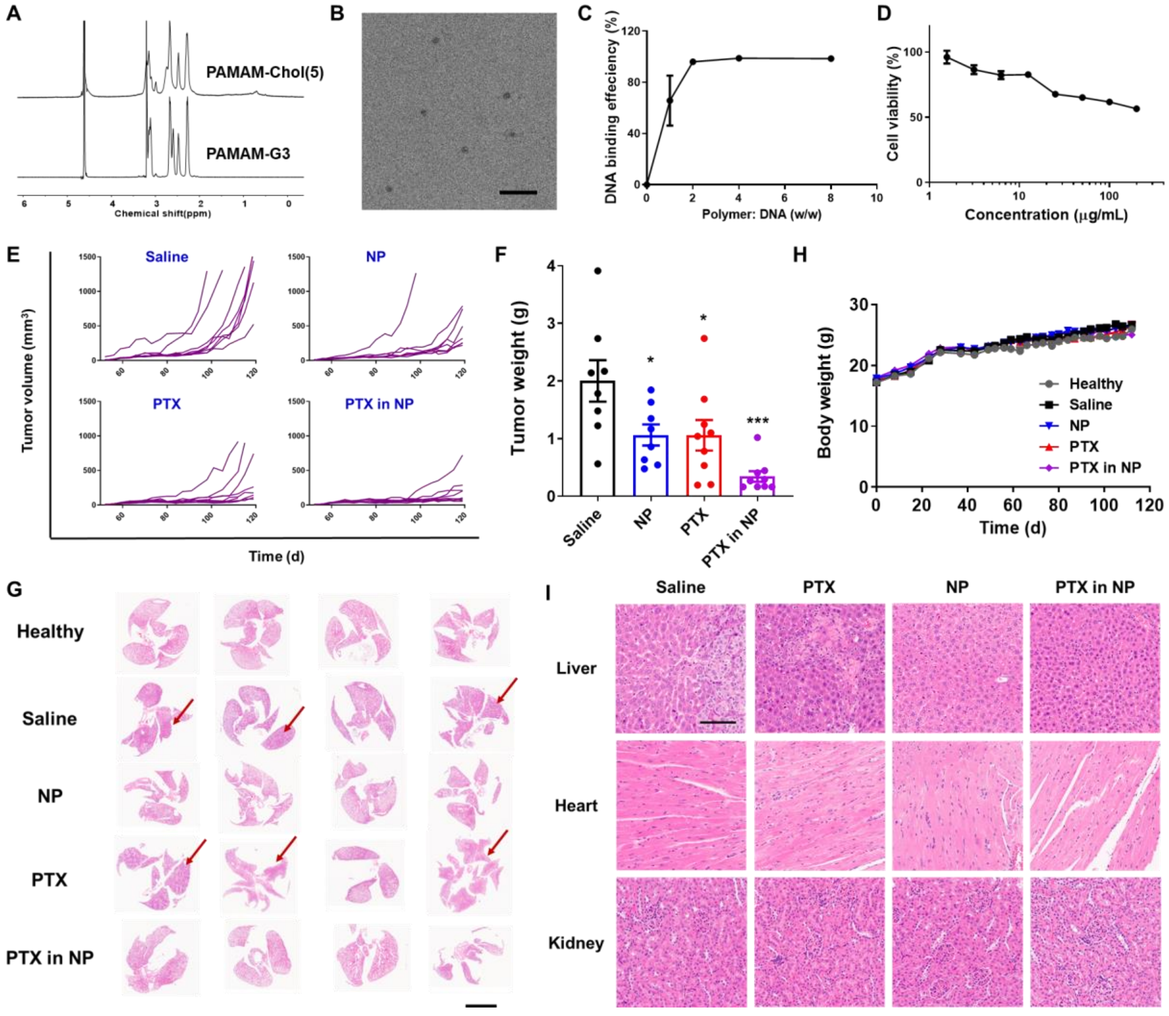

Fig. S7. Effect of PTX-loaded NPs in human breast cancer bearing NSG mouse model. (A) ^{1}H-NMR spectra of PAMAM-Chol(5) in comparison with PAMAM-G3. (B) TEM figure of PAMAM-Chol(5) NPs. Scale bar, 500 nm. (C) DNA binding efficiency of PAMAM-Chol(5) NPs at different polymer:DNA mass ratios. (D) Viability of MDA-MB-231 cells treated with PAMAM-Chol(5) NPs for 24 h. (E) Tumor growth curves for individual mice. (F) Tumor weights. Comparisons are with the saline-only group. **P*<0.05, ***P*<0.01, ****P*<0.001, *****P*<0.0001; Student's *t*-test. Data represent mean ± SEM. (G) H&E-stained lung sections for deep learning study. Red arrows indicate tumor metastasis. Scale bar, 6 mm. (H) Body weights of healthy and MDA-MB-231 mammary tumor-bearing NSG mice in different treatment groups. (I) H&E-stained sections of liver, heart and kidney showing no observable damage induced by the NPs. Scale bar, 50 μm.

Table S1. Properties of PAMAM dendrimer derivatives.

	Polymer	Number of terminal groups	Grafting groups	Number of grafting groups	% grafting	MW (Da)	DNA binding EC_{50} (Polymer:DNA)	Toxicity IC_{50} ($\mu g\ mL^{-1}$)
1	$G3\text{-}NH_2$	32	-	0	0	6910	0.67	31.6
2	$G3\text{-}DMEA_{24}$	32	DMEA	24	75.0%	9210	1.00	>500
3	$G3\text{-}DEEA_{10}$	32	DEEA	10	31.2%	8340	0.52	>500
4	$G3\text{-}DEEA_{24}$	32	DEEA	24	75.0%	9770	1.10	>500
5	$G4\text{-}NH_2$	64	-	0	0	14220	0.71	11.7
6	$G4\text{-}DMEA_{40}$	64	DMEA	40	62.5%	18820	1.04	>500
7	$G4\text{-}DMEA_{50}$	64	DEMA	50	78.1%	19970	0.83	>500
8	$G4\text{-}DEEA_{21}$	64	DEEA	21	32.8%	17220	0.83	19.5
9	$G4\text{-}DEEA_{40}$	64	DEEA	40	62.5%	19940	1.21	>500
10	$G4\text{-}DEEA_{50}$	64	DEEA	50	78.1%	21370	0.54	>500
11	$G4\text{-}DBEA_{20}$	64	DBEA	20	31.2%	18200	>10	50.5
12	$G4\text{-}DBEA_{40}$	64	DBEA	40	62.5%	21980	9.7	166.9
13	G2.5-COOH	32	-	0	0	6270	>10	>500
14	G3.5-COOH	64	-	0	0	12930	>10	>500

Table S2. Properties of nanoparticles fabricated from PAMAM dendrimer derivatives.

	Nanoparticle	Number of terminal groups	Grafting groups	Number of grafting groups	% grafting	MW (Da)	Diameter (nm)	Zeta potential (mV)
15	G3-$C12_5$-$DEEA_{20}$	32	DEEA, C12	25	78.1%	10,280	141.8 ± 3.3	58.2 ± 4.8
16	G3-$C12_9$-$DEEA_{20}$	32	DEEA, C12	29	90.6%	11,140	186.8 ± 5.6	55.0 ± 0.6
17	G4-$C12_7$-$DEEA_{35}$	64	DEEA, C12	42	65.6%	19,310	172.6 ± 0.4	62.6 ± 1.6
18	G4-$C12_9$-$DEEA_{35}$	64	DEEA, C12	44	68.8%	20,170	194.5 ± 2.4	60.8 ± 2.1
19	G4-$C12_{15}$-$DEEA_{35}$	64	DEEA, C12	50	78.1%	21,450	164.0 ± 9.5	65.4 ± 2.1

Table S3. Human primer sequences used for qPCR.

Primer name	Sequence (5' → 3')
hTLR3_FWD	CCT GGT TTG TTA ATT GGA TTA ACG A
hTLR3_RVRS	TGA GGT GGA GTG TTG CAA AGG
hTLR8_FWD	TTA TGT GTT CCA GGA ACT CAG AGA A
hTLR8_RVRS	TAA TAC CCA AGT TGA TAG TCG ATA AGT TTG
hTLR9_FWD	CCA CCC TGG AAG AGC TAA ACC
hTLR9_RVRS	GCC GTC CAT GAA TAG GAA GC
hIL-6_FWD	TCC ACA AGC GCC TTC GGT CCA
hIL-6_RVRS	AGG GCT GAG ATG CCG TCG AGG A
hTNF-alpha_FWD	CCT GTG AGG AGG ACG AAC AT
hTNF-alpha_RVRS	GGT TGA GGG TGT CTG AAG GA
hMYD88_FWD	CTC CTC CAC ATC CTC CCT TC
hMYD88_RVRS	CGC ACG TTC AAG AAC AGA GA
hNFkB p50_FWD	TGGACAGCAAATCCGCCCTG
hNFkB p50_RVRS	TGTTGTAATGAGTCGTCATCCT
hActin_FWD	ACC AAC TGG GAC GAC ATG GA
hActin_RVRS	CCA GAG GCG TAC AGG GAT AG
